# USP13 negatively regulates antiviral responses by deubiquitinating STING

**DOI:** 10.1038/ncomms15534

**Published:** 2017-05-23

**Authors:** He Sun, Qiang Zhang, Ying-Ying Jing, Man Zhang, Hai-Ying Wang, Zeng Cai, Tianzi Liuyu, Zhi-Dong Zhang, Tian-Chen Xiong, Yan Wu, Qi-Yun Zhu, Jing Yao, Hong-Bing Shu, Dandan Lin, Bo Zhong

**Affiliations:** 1 Department of Virology, College of Life Sciences, Wuhan University, Wuhan 430072, China; 2 Department of Immunology, Medical Research Institute, School of Medicine, Wuhan University, Wuhan 430071, China; 3 National Institute of Biological Sciences, Beijing 102206, China; 4 State Key Laboratory of Veterinary Etiological Biology, Lanzhou Veterinary Research Institute, Chinese Academy of Agricultural Sciences, Lanzhou 730046, China; 5 Cancer Center, Renmin Hospital of Wuhan University, Wuhan 430060, China

## Abstract

STING (also known as MITA) is critical for host defence against viruses and the activity of STING is regulated by ubiquitination. However, the deubiquitination of STING is not fully understood. Here, we show that ubiquitin-specific protease 13 (USP13) is a STING-interacting protein that catalyses deubiquitination of STING. Knockdown or knockout of USP13 potentiates activation of IRF3 and NF-κB and expression of downstream genes after HSV-1 infection or transfection of DNA ligands. USP13 deficiency results in impaired replication of HSV-1. Consistently, USP13 deficient mice are more resistant than wild-type littermates to lethal HSV-1 infection. Mechanistically, USP13 deconjugates polyubiquitin chains from STING and prevents the recruitment of TBK1 to the signalling complex, thereby negatively regulating cellular antiviral responses. Our study thus uncovers a function of USP13 in innate antiviral immunity and provides insight into the regulation of innate immunity.

## Abstract (web summary)

The cGAS-STING pathway is a DNA sensing mechanism that enables response to viral infection by inducing type 1 interferon expression. Here the authors show a mechanism by which the deubiquitinating enzyme USP13 prevents STING from enabling response to virus.

Innate immune system constitutes the first line for host defence against invading pathogens, which depends on germline-encoded pattern-recognition receptors that detect structurally conserved pathogen-associated molecular patterns (PAMPs) generated during the pathogen life cycle^1^. Some PAMPs, including lipopolysaccharides, peptidoglycans and flagellins, are exclusively found in pathogens, whereas others such as nucleic acids are found both in pathogen and in host where they are termed as danger-associated molecular patterns (DAMPs). However, because the modifications or locations of pathogenic nucleic acids are different from those of host, are essential PAMPs for many types of pathogen, especially viruses^2^. Viral DNA or RNA generated during infection and replication has long been recognized as a classical PAMP that is detected by nucleic acid sensors^3^. For example, cytoplasmic 5′ uncapped single-stranded RNA (ssRNA) or double-stranded RNA (dsRNA) is recognized by RIG-I-like receptors (RLRs) RIG-I and MDA5, whereas cytoplasmic DNA is recognized by a number of cytoplasmic DNA sensors, including DNA-dependent activator of IFN-regulatory factors (DAI), RNA polymerase III, interferon gamma inducible protein 16 (IFI16), DEAD-box helicase 41 (DDX41) and Lsm14A in cell-type or ligand-type dependent manners^4^,^5^,^6^,^7^,^8^,^9^. The nucleotidyl transferase cyclic GMP-AMP synthase (cGAS) is an important cytoplasmic sensor that recognizes various DNA ligands in a number of different cell types^10^,^11^,^12^.

On binding to viral nucleic acids, pattern-recognition receptors activate signalling cascades that lead to expression of hundreds of downstream genes, the products of which collaboratively inhibit viral replication and activate the adaptive immune responses. While RNA polymerase III transcribes AT-rich DNA into 5′pppRNA that activates RIG-I-MAVS signalling, other cytoplasmic DNA sensors such as DAI, IFI16, DDX41, Lsm14A and cGAS trigger signalling depending on the adaptor protein STING (also known as MITA, ERIS and MPYS)^13^,^14^,^15^,^16^. In particular, on binding to DNA, cGAS catalyses the synthesis of cGAMP which binds and triggers dimerization or oligomerization of STING^17^,^18^. Although STING primarily mediates innate immune signalling in response to DNA viruses, several studies have demonstrated that STING mediates RNA virus-triggered signalling and is required for defence against RNA viruses^13^,^14^,^15^,^19^,^20^. In addition to defence against viral infection, STING is also involved in autoimmunity in humans and mice^21^,^22^. Thus, it is conceivable that the activity of STING is tightly controlled to inhibit excessive autoimmunity and aberrant inflammation while facilitating defence against viruses.

The activity of STING is regulated by various ubiquitin modifications. For example, the E3 ubiquitin ligases TRIM56 and TRIM32 catalyse K63-linked ubiquitination of STING upon viral infection, which promotes the recruitment of TBK1 to STING and dimerization of STING^23^,^24^. RNF5 targets STING for K48-linked ubiquitination and proteasome-dependent degradation^25^, whereas RNF26 catalyses K11-linked ubiquitination of STING at the same lysine residue and thereby antagonizes RNF5-mediated K48-linked ubiquitination and degradation of STING^26^. A report has demonstrated that a viral infection-induced E3 ligase AMFR/gp78 interacts with STING constitutively and mediates K27-linked ubiquitination of STING, which is critical for STING-mediated recruitment of TBK1 and IRF3 after DNA virus infection^27^. However, the deubiquitinaiton of STING has not been investigated.

Ubiquitin-specific protease 13 (USP13) belongs to the deubiquitinating enzyme (DUB) superfamily and is implicated in tumorigenesis by deubiquitinating tumour suppressors p53, PTEN and MITF^28^,^29^,^30^. USP13 has been reported to deubiquitinate and stabilize STAT1 and promote interferon (IFN)-induced signalling^31^. USP13 has been shown to promote ERAD by antagonizing AMFR/gp78-mediated ubiquitination and proteolysis of Ubl4A, a central component of the Bag6 chaperon complex^32^. Whether USP13 deconjugates ubiquitin chains of other types of linkage from target proteins and how this process is related to a physiological significance is not clear.

Here, we perform an unbiased screen by coimmunoprecipitation assays in cells cotransfected with FLAG-tagged DUBs and HA-STING, and find that USP13 interacts with STING. While overexpression of USP13 inhibits virus-triggered induction of downstream genes, knockdown of USP13 or USP13 deficiency potentiates DNA virus-triggered activation of IRF3 and expression of type I IFNs and proinflammatory cytokines. Consistently, USP13-deficient mice restrict HSV-1 replication *in vivo* and are resistant to HSV-1 infection. USP13 deconjugates K27-linked polyubiquitin chains from STING, thereby inhibiting the recruitment of TBK1 to STING. Our findings thus reveal a previously uncharacterized role of USP13 by targeting STING in regulating innate immune signalling in response to DNA viruses.

## Results

### USP13 interacts with STING

**Figure 1:**
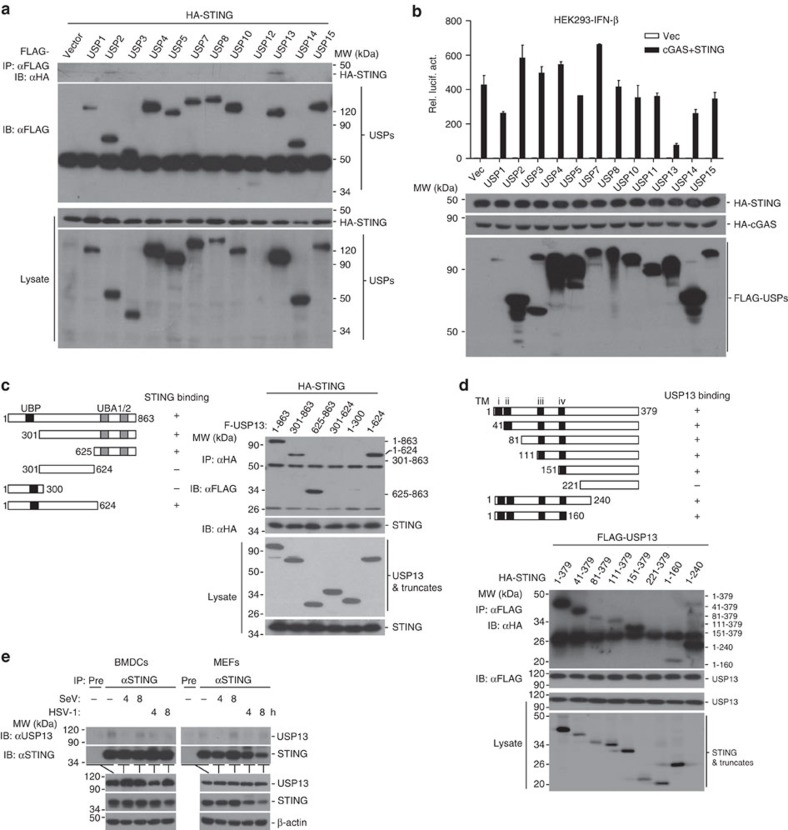
USP13 interacts with STING. (**a**) Immunoprecipitation (IP, with anti-FLAG) and immunoblot analysis (with anti-FLAG and anti-HA) of HEK293 cells transfected with plasmids encoding FLAG-tagged USPs and HA-STING for 24 h. (**b**) Luciferase reporter assays analysing IFN-β promoter activity (top) and immunoblot assay of FLAG-tagged USPs, Myc-STING and HA-cGAS of HEK293 cells transfected with the indicated plasmids for 24 h. (**c**,**d**) Immunoprecipitation (with anti-HA) and immunoblot analysis (with anti-FLAG or anti-HA) of HEK293 cells transfected with plasmids encoding HA-STING and FLAG-tagged USP13 or USP13 truncates for 24 h (**c**) or transfected with plasmids encoding FLAG-USP13 and HA-STING or STING truncates for 24 h (**d**). (**e**) Immunoprecipitation (with anti-STING or IgG as a control) and immunoblot analysis (with anti-STING or anti-USP13) of BMDCs (left) and MEFs (right). Data are representative of three independent experiments (mean±s.d. in **b**). See Supplementary Fig. 11 for uncropped immunoblots.

Previous studies have demonstrated that STING is modified by various types of ubiquitin chains which is essential for antiviral immunity^33^. We speculated that deubiquitination of STING is equally important as ubiquitination and thus screened the DUBs that interacted with STING and potentially catalysed deubiquitination of STING. This effort led to the identification of USP13, as a STING-interacting protein (Fig. 1a). In a parallel reporter screen, USP13 inhibited cGAS and STING-induced activation of IFN-β promoter (Fig. 1b). In contrast, USP5 which shares 80% sequence similarity with USP13, neither interacted with STING in an overexpression system nor inhibited cGAS and STING-induced activation of IFN-β promoter in reporter assays (Fig. 1a,b), indicating a specific interaction between USP13 and STING.

We next mapped the domains mediating STING-USP13 association and found that the transmembrane domains of STING and an intact N-terminal (aa1–624) or C-terminal (aa625–863) domain of USP13 were responsible for their association (Fig. 1c,d), indicating that the structural basis of multiple domains of USP13 (either aa1–624 or aa625–863) but not the UBP domain or the isopeptidase domain is the primary determinant for its association with STING. Results from endogenous immunoprecipitation and immunoblot analysis revealed that USP13 constitutively interacted with STING without viral infection in mouse bone marrow-derived dendritic cells (BMDCs) and mouse embryonic fibroblasts (MEFs) (Fig. 1e). Interestingly, SeV infection transiently disrupted USP13-STING association, whereas HSV-1 infection impaired but not totally abolished the interaction between USP13 and STING (Fig. 1e). Immunoflorescence and confocal microscopy analysis showed that a substantial portion of USP13 was colocalized with the ER marker Rab9. In contrast, little USP13 was colocalized with the autophagy marker Atg12 or the mitochondrial marker COX4. In addition, the colocalization of USP13 and Rab9 was not affected by HSV-1 infection (Supplementary Fig. 1a). We also observed that a portion of USP13 was colocalized with STING at the ER without infection and HSV-1 infection resulted in STING disassociation from USP13 and redistribution in the cytoplasm (Supplementary Fig. 1b), consistent with previous observations that STING migrates out of ER after HSV-1 infection^34^. These data together suggest that USP13 interacts with STING under physiological conditions.

### USP13 inhibits HSV-1-triggered signalling in human cells

**Figure 2:**
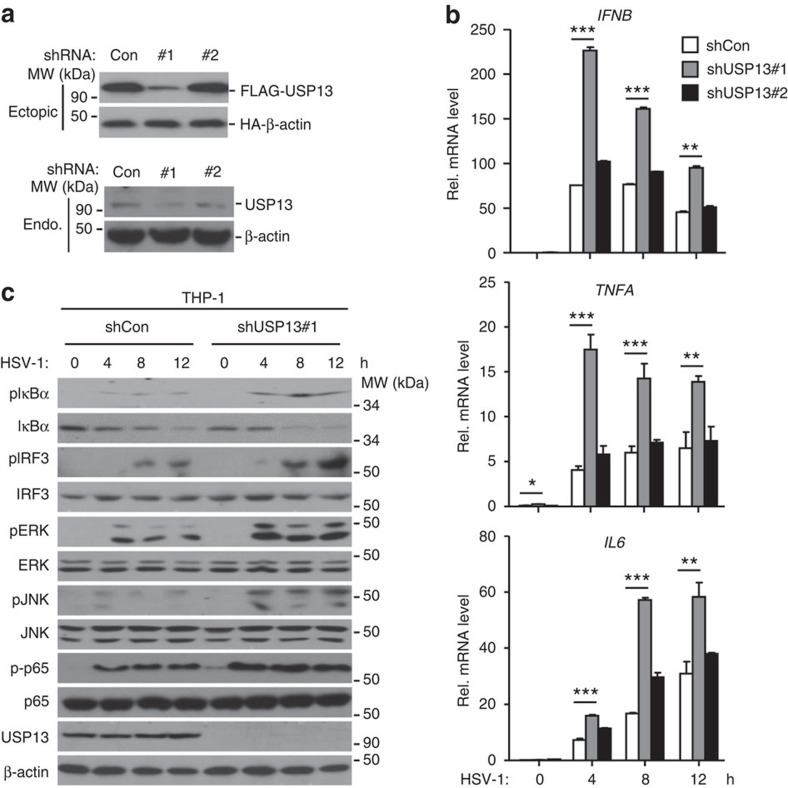
USP13 restricts HSV-1-triggered signalling in THP-1 cells. (**a**) Immunoblot analysis (with anti-FLAG or anti-HA) of HEK293 cells transfected for 36 h with plasmids encoding FLAG-tagged USP13 and HA-β-Actin and either USP13-targeting shRNA (shUSP13#1 and shUSP13#2) or control shRNA (Con) to test efficiency of shRNA (upper panels). Immunoblot analysis (with anti-USP13 or anti-β-Actin) of THP-1 cells stably transfected with plasmids encoding control (Con) shUSP13 (#1 or #2) (lower panels). (**b**) qPCR analysis of *IFNB*, *TNFA* or *IL6* mRNA in THP-1 cells stably transfected with shCon, shUSP13#1 or shUSP13#2 followed by infection with SeV or HSV-1 for 0–12 h. (**c**) Immunoblot analysis of phosphorylation of IRF3 and IκBα, ERK, JNK, or total IRF3 and IκBα, ERK, JNK, USP13 and β-Actin THP-1 cells stably transfected with shCon or shUSP13#1 followed by infection with HSV-1 for 0–12 h. **P<0.01; ***P<0.001 (analysis of two-way ANOVA followed by Bonferroni post-test). Data are representative of three independent experiments (mean±s.d. in **b**). See Supplementary Fig. 12 for uncropped immunoblots.

USP13 has been implicated in ER-associated degradation (ERAD), tumorigenesis, and type I IFN-triggered signalling by deubiquitinating a series of proteins^28^,^29^,^30^,^32^. Since USP13 interacts with STING, an adaptor protein critically involved in innate antiviral responses, we investigated the role of USP13 in virus-triggered signalling in THP-1 cells by shRNA-mediated knockdown of USP13 (Fig. 2a). The results showed that HSV-1- but not SeV-triggered expression of *IFNB*, *TNFA* and *IL6* was enhanced in THP-1 cells stably transfecting with shRNA targeting USP13 (Fig. 2b and Supplementary Fig. 2a). In addition, knockdown of USP13 by shRNA potentiated HSV-1- but not SeV-induced phosphorylation of IRF3, IκBα and MAPKs including ERK and JNK in THP-1 cells (Fig. 2c and Supplementary Fig. 2b), indicating that USP13 negatively regulates HSV-1-triggered signalling in THP-1 cells.

To substantiate these observations, we examined the effects of siRNA-mediated knockdown of USP13 on virus-triggered signalling. As shown in Supplementary Fig. 2c, two of three siRNAs targeting USP13 severely impaired endogenous expression of USP13 (The #2 siRNA was used for the experiments described below and similar results were obtained with the #1 siRNA.). Knockdown of USP13 by transiently transfecting with siUSP13 significantly potentiated expression of HSV-1-, but not SeV-triggered expression of *IFNB* and *TNFA* in THP-1 cells (Supplementary Fig. 2d). Consistent with the results from gene induction assays, knockdown of USP13 by siRNA promoted HSV-1- but not SeV-induced phosphorylation of IRF3 and IκBα in THP-1 cells (Supplementary Fig. 2e,f). These data together suggest that USP13 negatively regulates DNA viruses-triggered signalling in human cell lines.

### USP13 restricts DNA virus-triggered signalling in mouse cells

To further investigate the role of USP13 in antiviral signalling, we generated USP13-deficient mice by CRISPR/Cas9-mediated genome editing (Supplementary Fig. 3a). Results from sequencing showed that a single cytosine was inserted into the first exon of *Usp13* gene at the 97 position of the *Usp13* open reading frame, which caused a frame shift and led to an early translational termination of USP13 (aa1–57) (Supplementary Fig. 3b). Immunoblot analysis further demonstrated a complete loss of USP13 protein in the *Usp13*^m/m^ mice (Supplementary Fig. 3c). The *Usp13*^+/m^ mice bred normally with the Mendelian inheritance ratio and the *Usp13*^m/m^ mice did not show any developmental defect until 12-week old compared to the wild-type littermates (Supplementary Fig. 4a,b). The differentiation of *Usp13*^m/m^ bone marrow cells into BMDC, BMDM or pDCs was similar to that of wild-type counterparts in the presence of GM-CSF, M-CSF or Flt3L, respectively (Supplementary Fig. 4c). The numbers and percentages of various immune cells in thymus, spleen or peripheral lymph nodes were comparable between the wild-type and *Usp13*^m/m^ mice (Supplementary Fig. 4d–f), indicating that USP13 is dispensable for development and homoeostasis of immune cells.

**Figure 3:**
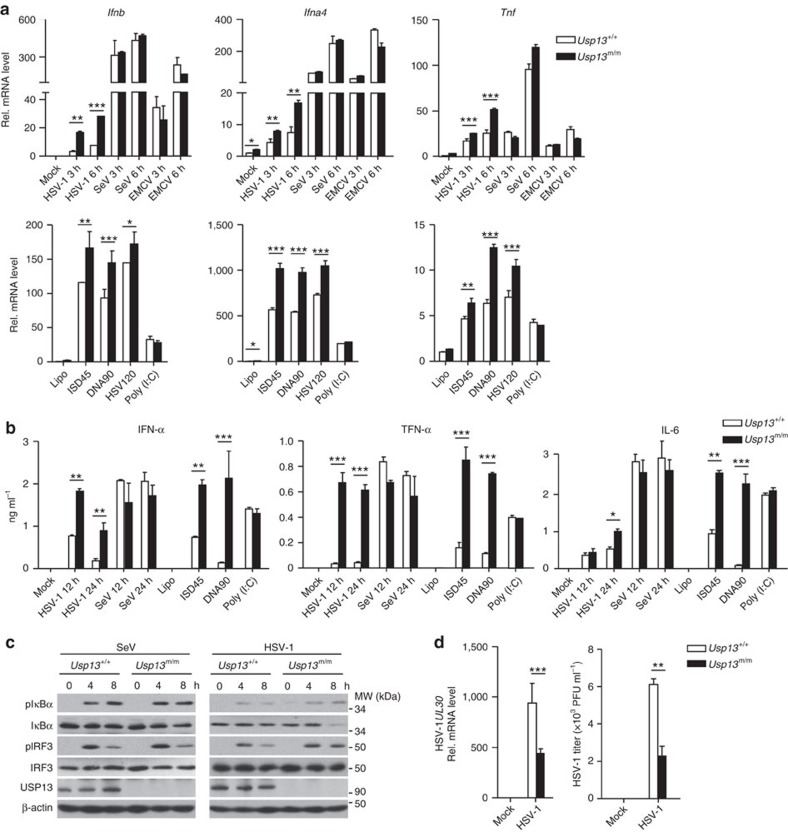
USP13 deficiency potentiates DNA virus-triggered signalling. (**a**) qRT-PCR analysis of *Ifnb*, *Ifna4*, and *Tnf* mRNA in *Usp13*^+/+^ and *Usp13*^m/m^ BMDCs left uninfected (Mock) or infected with HSV-1, SeV or EMCV for 3–6 h or mock transfected (Lipo) or transfected with ISD45, DNA90, HSV120 or poly(I:C) for 6 h. (**b**) ELISA analysis of IFN-α, TNFα and IL-6 in the supernatants of *Usp13*^+/+^ and *Usp13*^m/m^ BMDC infected for 12–24 h, or mock transfected (Lipo) or transfected with ISD45, DNA90 or poly(I:C) for 12 h. (**c**) Immunoblot analysis of phosphorylation of IRF3 and IκBα, total IRF3 and IκBα, USP13 and β-Actin in *Usp13*^+/+^ and *Usp13*^m/m^ BMDCs infected with SeV or HSV-1 for 0–8 h. (**d**) qPCR analysis of HSV-1 *UL30* mRNA in *Usp13*^+/+^ and *Usp13*^m/m^ BMDCs (5 × 10^5^) left uninfected or infected with HSV-1 (MOI=0.1) for 1 h followed by twice PBS wash and cultured in full medium for 24 h (left graph). Plaque assay of HSV-1replication in the supernatants of *Usp13*^+/+^ and *Usp13*^m/m^ BMDCs infected with HSV-1 (MOI=0.1) for 1 h followed by twice PBS wash and cultured in full medium for 36 h (right graph). *P<0.05; **P<0.01; ***P <0.001 (analysis of two-way ANOVA followed by Bonferroni post-test). PFU, plaque-forming unit. Data are representative of three independent experiments (mean±s.d. in **a**,**b**,**d**). See Supplementary Fig. 12 for uncropped immunoblots.

We next examined virus-triggered induction of downstream genes in *Usp13*^+/+^ and *Usp*13^m/m^ cells. Interestingly, HSV-1- or cytoplasmic DNA- but not SeV-, EMCV- or transfected poly(I:C)-induced expression of *Ifnb*, *Ifna4* or *Tnf* and the production of IFN-α, TNF or IL-6 were enhanced in *Usp*13^m/m^ BMDCs compared to the *Usp*13^+/+^ BMDCs (Fig. 3a,b). Similarly, HSV-1-triggered induction of *Ifnb*, *Ifna4*, *Tnf*, or *Ccl5* was increased in *Usp*13^m/m^ MEFs or BMDMs compared to the *Usp*13^+/+^ counterparts (Supplementary Fig. 5a). Consistent with these observations, HSV-1- but not SeV-induced phosphorylation of IRF3 and IκBα was increased in *Usp*13^m/m^ BMDCs and MEFs compared to the *Usp*13^+/+^ counterparts (Fig. 3c and Supplementary Fig. 5b). Moreover, the replication of HSV-1 or HSV-1-GFP was suppressed in *Usp*13^m/m^ BMDCs or MEFs compared to the *Usp*13^+/+^ counterparts as monitored by the expression of HSV-1 *UL30* gene, the GFP percentages of HSV-1-GFP or the HSV-1 titres in the supernatants (Fig. 3d and Supplementary Fig. 5c–e). These data together suggest that USP13 negatively regulates DNA virus-triggered signalling in various primary mouse cells.

### USP13 deficient mice are more resistant to HSV-1 infection

**Figure 4:**
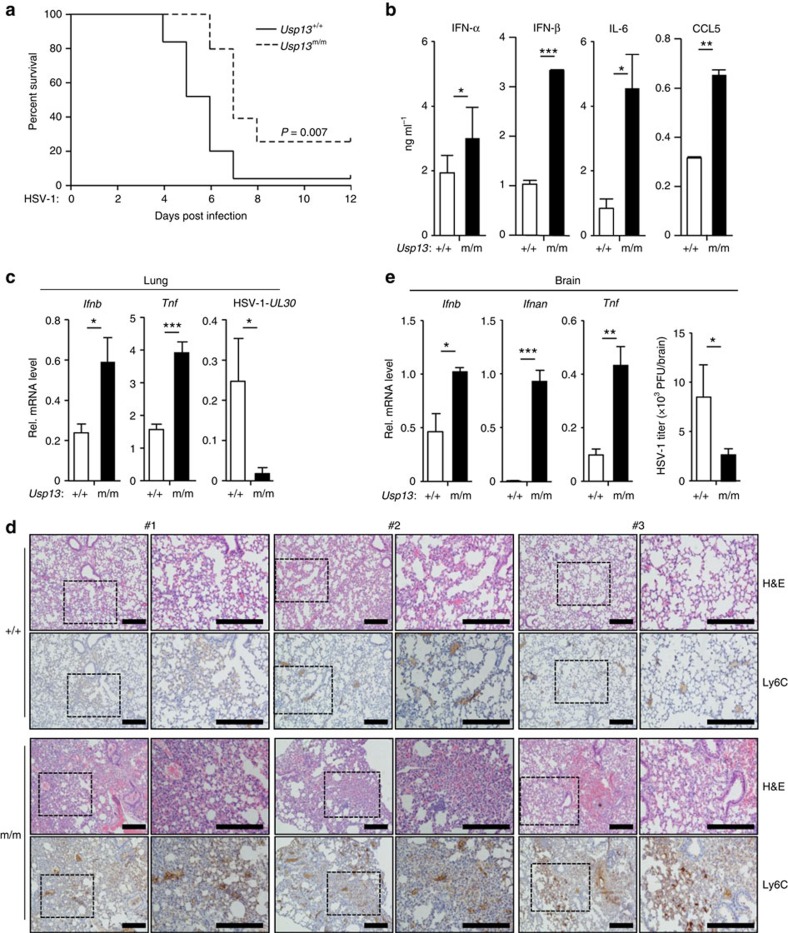
USP13 deficient mice are more resistant to HSV-1 infection. (**a**) Survival (Kaplan-Meier curve) of *Usp13*^+/+^ (*n*=19) and *Usp13*^m/m^ mice (*n*=16) intravenously injected with HSV-1 (2.5 × 10^6^ PFU per mouse) monitored survival for 12 days. (**b**) ELISA analysis of IFN-α, IFN-β, IL-6 and CCL5 in the sera of *Usp13*^+/+^ and *Usp13*^m/m^ mice (*n*=5) intravenously injected with HSV-1 (2.5 × 10^6^ PFU per mouse) for 12 h. (**c**,**d**) qRT-PCR analysis of *Ifnb*, *Tnf*, or HSV-1 *UL30* mRNA in the lungs (**c,** *n*=5) or histological analysis (H&E staining or Ly6C staining) of lung sections (**d,** *n*=3) from *Usp13*^+/+^ and *Usp13*^m/m^ mice intravenously injected with HSV-1 (2.5 × 10^6^) for 24 h. (**e**) qRT-PCR analysis of *Ifnb*, *Ifna*, and *Tnf* and plaque assays of the brains from *Usp13*^+/+^ and *Usp13*^m/m^ mice (*n*=5) intraperitoneally injected with HSV-1 (5 × 10^6^) for 4 days. *P<0.05; **P<0.01; ***P<0.001 (unpaired student's *t*-test, **b**,**c**,**e**). Scale bars represent 100 μM. PFU, plaque-forming unit. Data are of combined three independent experiments (**a**) or representative of two (**b**–**e**) independent experiments (mean±s.d. in **b**,**c**,**e**).

To gain insight into the function of USP13 in host defence against viral infection *in vivo*, we monitored survival of *Usp13*^+/+^ and *Usp*13^m/m^ mice after intravenous (i.v.) injection of HSV-1. *Usp*13^m/m^ mice exhibited a later onset of death (day 6 versus day 4 after infection) and a higher survival rate (4/16 versus 1/19, *P*=0.007) compared to the wild-type littermates (Fig. 4a). The concentrations of IFN-α, IFN-β, IL-6 and CCL5 proteins were significantly increased in sera from *Usp*13^m/m^ mice compared to those in sera from the wild-type littermates at 12 h after HSV-1 infection (Fig. 4b). The expression of *Ifnb* and *Tnf* was potentiated and the expression of HSV-1 *UL30* gene was inhibited in the lungs from *Usp13*^m/m^ mice compared to *Usp13*^+/+^ mice at 24 h after HSV-1 infection (Fig. 4c). Consistent with these observations, hematoxylin and eosin staining and immunohistochemistry analysis of lung tissues showed that more Ly6C^+^ monocytes existed in the lungs of *Usp13*^m/m^ mice than in the lungs of wild-type mice at 24 h after HSV-1 infection (Fig. 4d). We analysed the expression of cytokines and viral titres in the brains 4 days after HSV-1 infection and observed that the expression of *Ifnb*, *Ifna* and *Tnf* was increased and the replication of HSV-1 was suppressed in the brains from *Usp13*^m/m^ mice compared to those from *Usp13*^+/+^ mice (Fig. 4e). Collectively, these data suggest that loss of USP13 protects mice from HSV-1 infection by promoting the induction of type I IFNs and proinflammatory cytokines.

### The DUB activity of USP13 is required for suppression

**Figure 5:**
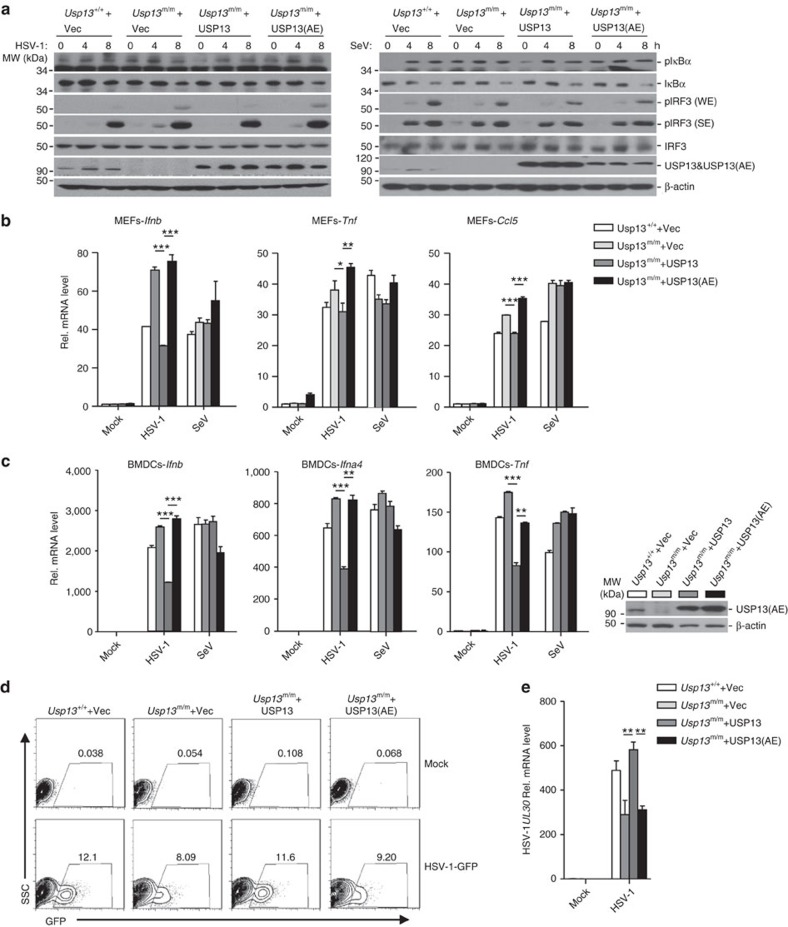
Suppression of antiviral signalling by USP13 requires its DUB activity. (**a**,**b**) Immunoblot analysis of phosphorylation of IRF3 and IκBα, total IRF3 and IκBα, USP13 and β-Actin (**a**), and qRT-PCR analysis of *Ifnb*, *Tnf*, or *Ccl5* mRNA (**b**) in *Usp13*^+/+^ MEFs reconstituted with the empty vector (*Usp13*^+/+^+Vec) or in *Usp13*^m/m^ MEFs reconstituted with the empty vector (*Usp13*^m/m^+Vec), USP13 (*Usp13*^m/m^+USP13), or USP13 (C345A/M664/739E) (*Usp13*^m/m^+USP13(AE)) infected with HSV-1 or SeV for 0–8 or 6 h. (**c**,**d**) Immunoblot (**c**, right panels), qRT-PCR (**c**, left graphs) and flow cytometry analysis (**d**) of *Usp13*^+/+^+Vec, *Usp13*^m/m^+Vec, *Usp13*^m/m^+USP13, and *Usp13*^m/m^+USP13(AE) BMDCs infected with HSV-1 or SeV (**c**) for 6 h or HSV-1-GFP (**d**) for 24 h. Numbers adjacent to the outlined areas indicate percentages of GFP^+^ BMDCs. (**e**) qRT-PCR analysis of HSV-1 *UL30* gene in cells obtained in (**c**) infected with HSV-1 for 24 h. *P<0.05; **P<0.01; ***P<0.001 (analysis of two-way ANOVA followed by Bonferroni post-test). USP13(AE): USP13(C345A/M664/739E). Data are representative of two (**a**) or three (**b**–**e**) independent experiments (mean±s.d. in **b**,**c**,**e**). See Supplementary Fig. 12 for uncropped immunoblots.

We next examined whether the deubiquitinating activity (DUB) was required for USP13-mediated suppression of antiviral signalling. The empty vector, USP13 or its enzymatic inactive mutant USP13 (C345A/M664/739E) (designated as USP13(AE)) was reconstituted into *Usp13*^m/m^ cells followed by SeV or HSV-1 infection. As shown in Fig. 5a, HSV-1- but not SeV-induced phosphorylation of IRF3 or IκBα was impaired by reconstitution of USP13 but not USP13(AE) into *Usp13*^m/m^ MEFs. Results from quantitative real-time PCR (qRT-PCR) analysis showed that HSV-1- but not SeV-induced expression of *Ifnb*, *Ifna4*, *Ccl5* or *Tnf* was substantially inhibited in *Usp13*^m/m^ MEFs or BMDCs reconstituted with USP13 but not in those reconstituted with USP13(AE) (Fig. 5b,c). Consistently, replication of HSV-1 was potentiated in *Usp13*^m/m^ BMDCs reconstituted with USP13 but not in those reconstituted with USP13(AE) as monitored by GFP signals or the expression of HSV-1 *UL30* gene (Fig. 5d,e). These data suggest that USP13-mediated suppression of HSV-1-triggered signalling requires its deubiquitinating enzymatic activity.

### USP13 targets STING for deubiquitination

**Figure 6:**
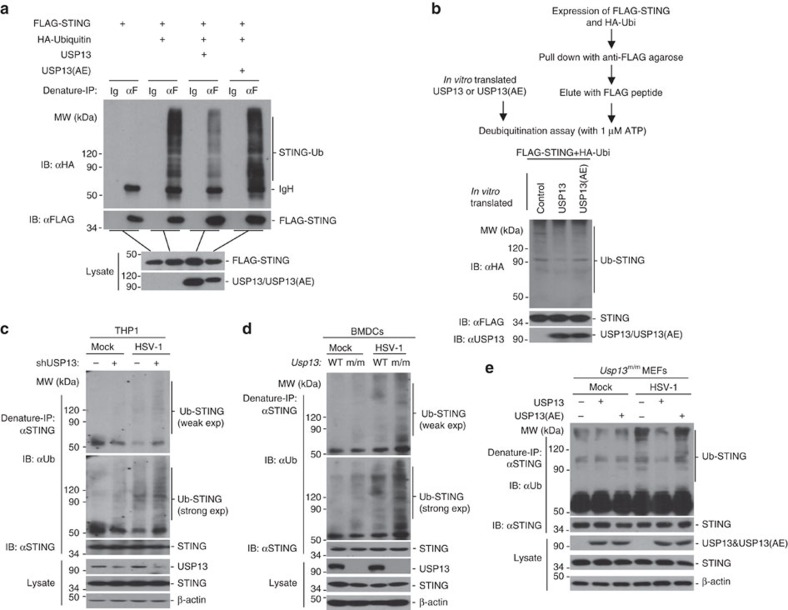
USP13 targets STING for deubiquitination. (**a**) Denature-immunoprecipitation (Denature-IP) (with anti-FLAG or IgG as a control) and immunoblot analysis (with anti-FLAG, anti-HA or anti-USP13) of HEK293 cells transfected with plasmids encoding FLAG-MITA, HA-Ubiquitin and empty vector, USP13 or USP13(AE) for 24 h. (**b**) The strategy for *in vitro* deubiquitination assay (upper scheme). *In vitro* deubiquitination analysis of ubiquitin-modified STING eluted from anti-FLAG precipitates by FLAG peptide of HEK293 cells transfected with FLAG-STING and HA-ubiquitin incubated with *in vitro* generated USP13 or USP13(AE) obtained from an *in vitro* transcription and translation kit. (**c**,**d**) Immunoprecipitation (with anti-STING) and immunoblot analysis (with anti-ubiquitin, anti-STING, anti-USP13 or anti-β-Actin as a control) of THP-1 cells stably transfected with shUSP13 or Con (**c**) or *Usp13*^+/+^ and *Usp13*^m/m^ BMDCs (**d**) left uninfected or infected with HSV-1 for 8 h. (**e**) Denature-IP (with anti-STING) and immunoblot analysis (with anti-Ub, anti-STING, anti-USP13 or anti-β-Actin as a control) of *Usp13*^m/m^ MEFs reconstituted with empty vector, USP13, or USP13(AE) left uninfected or infected with HSV-1 for 8 h. USP13(AE): USP13(C345A/M664/739E). Data are representative of at least three independent experiments. See Supplementary Fig. 13 for uncropped immunoblots.

Since USP13 is a STING-interacting DUB and its deubiquitinating enzymatic activity is required for regulating antiviral signalling, we investigated whether USP13 eliminated polyubiquitin chains from STING. As expected, we found that USP13 but not the enzymatic inactive mutant USP13(AE) catalysed deubiquitination of STING in cells or *in vitro* (Fig. 6a,b). In contrast, USP13 did not disrupt the association between STING and AMFR or TRIM32, two E3s catalyzing K27- or K63-linked ubiquitination of STING, respectively (Supplementary Fig. 6a). Although USP13 interacted with cGAS and IRF3 in an overexpression system, USP13 did not affect the ubiquitination of cGAS or IRF3 when coexpressed in HEK293 cells (Supplementary Fig. 6b,c). In addition, overexpression of USP13 but not USP13(AE) substantially inhibited STING- or cGAS plus STING- but not TBK1- or IRF3-mediated activation of ISRE, whereas knockdown of USP13 potentiated STING- or TBK1- but not IRF3-mediated activation of ISRE (Supplementary Fig. 6d), supporting the notion that USP13 suppresses antiviral signalling by targeting STING.

We have observed that the intact N-terminal (aa1-624) or C-terminal (625–863) domain interacted with STING (Fig. 1c). However, neither of them inhibited cGAS and STING-mediated activation of IFN-β promoter or deubiquitinated STING (Supplementary Fig. 7a,b). In contrast, the truncate (aa301-863) that preserves DUB activity and UBA motifs inhibited cGAS and STING-mediated activation of IFN-β promoter and catalysed deubiquitination of STING (Supplementary Fig. 7), indicating that USP13-mediated deubiquitination of STING is associated with its suppression of antiviral signalling.

We next examined virus-triggered ubiquitination of STING in the absence of USP13. The basal ubiquitination of STING was increased in USP13-knockdown THP-1 cells or in USP13 deficient BMDCs or MEFs compared to the controls without viral infection (Fig. 6c,d and Supplementary Fig. 8a,c). In addition, HSV-1-infection enhanced ubiquitination of STING more profoundly in USP13-knockdown THP-1 cells or in USP13 deficient BMDCs or MEFs compared to the controls (Fig. 6c,d and Supplementary Fig. 8b,c). In contrast, SeV-induced ubiquitination of STING was comparable between USP13-sufficient and deficient cells (Supplementary Fig. 8d,e). Consistently, reconstitution of USP13 but not USP13(AE) into *Usp13*^m/m^ MEFs inhibited HSV-1-induced ubiquitination of STING (Fig. 6e). Taken together, these data suggest that USP13 targets STING for deubiquitination in the presence or absence of viral infection.

### USP13 impairs the recruitment of TBK1 to STING

**Figure 7:**
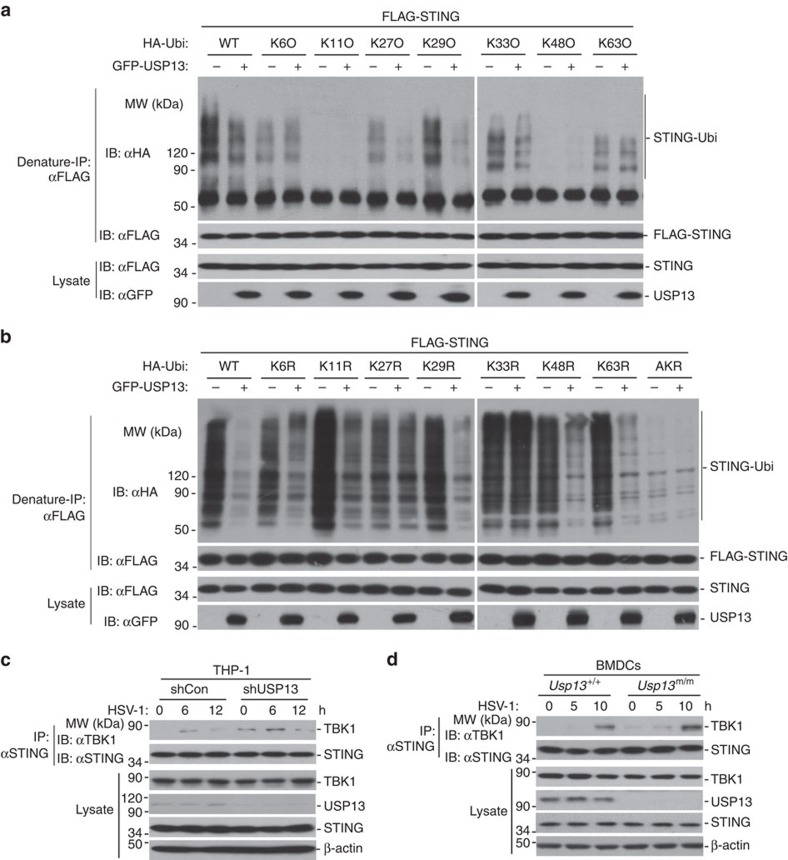
USP13 impairs the recruitment of TBK1 to STING. (**a**,**b**) Denature-IP (with anti-FLAG) and immunoblot analysis (with anti-FLAG, anti-HA or anti-GFP) of HEK293 cells transfected with plasmids encoding FLAG-STING, HA-tagged ubiquitin or mutations and either the empty vector or GFP-USP13 for 24 h. (**c**,**d**) Immunoprecipitation (with anti-STING) and immunoblot analysis (with anti-TBK1, anti-STING, anti-USP13 or anti-β-Actin) of THP-1 cells stably transfected with shUSP13 or shCon infected with HSV-1 for 0–12 h (**c**) or *Usp13*^+/+^ and *Usp13*^m/m^ BMDCs infected with HSV-1 for 0–10 h (**d**). Data are representative of three independent experiments. See Supplementary Fig. 13 for uncropped immunoblots.

It has been reported that K27- or K63-linked ubiquitination of STING is essential for mediating antiviral signalling. To test whether USP13 removed K27- or K63-linked polyubiquitin chains from STING, we transfected STING together with ubiquitin, or ubiquitin mutants either retaining a single lysine residue (KO) or retaining all but one lysine residues (KR) in the presence or absence of USP13 followed by deubiquitination analysis. The results showed that USP13 catalysed removal of K27O- or K33O-linked but not K27R-linked polyubiquitin chains from STING in cells or *in vitro* (Fig. 7a,b and Supplementary Fig. 9a). In contrast, USP13 deconjugated K63R- but not K63O-linked ubiquitination of STING (Fig. 7a,b). USP13 also weakly deconjugated K29O-linked ubiquitination of STING in cells (Fig. 7a). However, USP13 did not remove K29O-linked polyubiquitin chains from STING *in vitro* (Supplementary Fig. 9a). In addition, we observed that knockdown of AMFR/gp78 significantly dampened the potentiation of induction of downstream genes in *Usp13*^m/m^ MEFs after HSV-1 infection (Supplementary Fig. 9b). These data together suggest that USP13 primarily removes K27- but not K63- or K29-linked ubiquitination of STING.

Because K27-linked ubiquitination of STING mediates the recruitment of TBK1, we investigated the effects of USP13 on STING-TBK1 interaction. Interestingly, overexpression of USP13 inhibited TBK1-STING association but not TBK1-MAVS association (Supplementary Fig. 9c). In contrast, knockdown of USP13 in THP-1 cells or USP13 deficiency in BMDCs potentiated the recruitment of TBK1 to STING in the presence or absence of HSV-1 infection (Fig. 7d,e). These data together suggest that USP13 inhibits the recruitment of TBK1 to STING by deubiquitinating STING.

## Discussion

STING is an essential adaptor protein for innate antiviral immunity and fully activation of STING requires K27- or K63-linked ubiquitination mediated by various E3 ubiquitin ligases^23^,^24^,^27^. In this study, we found that USP13 deconjugated K27-linked polyubiquitin chains from STING and thereby impaired the recruitment of TBK1 to turn down the antiviral immune response against DNA viruses. In support of this notion, we observed that (i) USP13 deficiency in mice or knockdown of USP13 in human cells potentiated HSV-1-triggered activation of IRF3 and NF-κB and subsequent induction of type I IFNs and proinflammatory cytokines, (ii) USP13 deficient cells restricted HSV-1 replication more profoundly than did the wild-type controls and USP13 deficient mice were more resistant to lethal HSV-1 infection compared to the wild-type littermates, (iii) USP13 but not USP13(AE) removed K27-linked ubiquitination of STING in cells or *in vitro*, and (iv) overexpression of USP13 specifically disrupted TBK1-STING association and knockdown or knockout of USP13 potentiated STING-TBK1 association after HSV-1 infection. These findings thus uncover a new regulatory role of USP13 in innate antiviral signalling.

USP13 is an ER-associated DUB that promotes the ERAD process by antagonizing AMFR/gp78-mediated ubiquitination of Ubl4A, an essential component of the Bag6 chaperone complex^32^. In our study, we found that a portion of USP13 localized to the ER and constitutively interacted with STING in uninfected cells. Consistent with these observations, the basal ubiquitination of STING, interaction between TBK1 and STING, and the expression of several downstream genes were increased in USP13-knockdown THP-1 cells or in USP13 deficient cells compared to the controls without viral infection, indicating that USP13 keeps the innate immune signalling in check in unstimulated cells. In this context, it has been reported that the E3 ligase AMFR/gp78 constitutively interacts with and mediates basal K27-linked ubiquitination of STING in the absence of viral infection^27^. In contrast to STING translocating from ER to form puncta and recruit TBK1, the distribution of USP13 in cells was not affected by HSV-1 infection, indicating that HSV-1-induced redistribution of STING results in dissociation from USP13. Results from endogenous immunoprecipitation and immunofluorescence and microscopy analysis showed that a small portion of USP13 still interacted with STING on ER after HSV-1 infection. Indeed, HSV-1-induced activation of IRF3 and NF-κB and subsequent induction of downstream genes were enhanced in USP13 deficient mouse cells or in USP13-knockdown THP-1 cells, suggesting that the residual USP13-STING association restricts excessive activation of STING in response to viral infection. Consistent with these observations, deficiency of USP13 in mice resulted in increased ubiquitination of STING, production of type I IFNs and proinflammatory cytokines and exacerbated lung inflammation after HSV-1 infection. Thus, it is likely that USP13 plays a dual role under physiological and infectious conditions by keeping basal immune response in check and inhibiting virus-triggered excessive harmful inflammation and immunity, respectively.

The *Usp13*^m/m^ mice developed normally and did not show any autoimmune diseases until 10-month old. Whether the autoimmune symptoms will be observed in older *Usp13*^m/m^ mice remains to be explored. It should be noted that although basal ubiquitination of STING was increased in the absence of USP13, only weak (if any) increase of basal expression of selected genes was observed in these cells compared to the controls. Considering that fully activation of STING requires cGAMP binding- and ubiquitination-mediated oligomerization, the increased basal ubiquitination of STING alone without challenge by DNA viruses or cytoplamic DNA ligands might not be sufficient for full activation of STING and sustained activation of downstream genes. Alternatively, the increased basal ubiquitination of STING might induce basal expression of negative regulators of immune activation which inhibits overactivation of basal immune responses in *Usp13*^m/m^ cells or mice. In this context, it has been demonstrated that loss of STING which causes suppression of negative regulators involved in immune responses renders mice more susceptible to systemic autoimmunity in the mouse lupus model^35^.

Our results also suggested that USP13 deconjugated K33-linked ubiquitination of STING in cells and *in vitro*. It is unclear how the K33-linked ubiquitination of STING is mediated and whether it is related to the physiological function of STING. Further investigations are required to address this point. Although USP13 deconjugated K29O-linked ubiquitin chains from STING in cells, USP13 failed to do so *in vitro*. The reason behind the phenomenon is currently unknown. A possible explanation for this is that USP13 might remove K29-linked ubiquitination of STING together with other DUBs. In this context, it has been demonstrated that USP13 recruits and deubiquitinates USP10 which is essential for deubiquitination and stabilization of Beclin1 and our recent study has identified multiple DUBs interacting with STING^28^,^36^. Future studies will focus on whether and how the STING-interacting DUBs and USP13 cooperate to deubiquitinate K29-linked ubiquitination of STING. Nonetheless, our study has clearly demonstrated that USP13 directly deconjugates polyubiquitin chains from STING and thereby prevent the recruitment of TBK1 to restrict antiviral responses.

Based on our data and others, we proposed a working model on USP13-medaited regulation of STING during innate antiviral signalling (Supplementary Fig. 10). In unstimulated cells, USP13 interacts with and catalyses deubiquitination of STING which constitutively undergoes K27-linked ubiquitination mediated by AMFR/gp78, thereby restricting the basal immune signalling. Upon infection with DNA viruses, the majority of USP13-interacting STING is disassociated from USP13 and translocates to form puncta, thereby leading to increased ubiquitination of STING and boosted recruitment of TBK1. Meanwhile, a portion of USP13 still interacts with STING to prevent excessive immune and inflammatory responses. Our study contributes to the complicated regulatory molecular mechanisms of antiviral responses and provides USP13 as a potential target for future adjuvant or medicine design for infectious diseases.

## Methods

### Mice

The *Usp13*^m/m^ mice were generated by the CRISPR/Cas9-mediated genome editing. In brief, the vectors encoding Cas9 (44758, Addgene) and guide RNA were *in vitro* transcribed into messenger RNA (mRNA) and gRNA followed by injection into the fertilized eggs that were transplanted into pseudopregnant mice. The targeted genome of F0 mice was amplified with PCR and sequenced and the chimeras were crossed with wild-type C57BL/6 mice to obtain the *Usp13*^+/m^ mice. The F1 *Usp13*^+/m^ mice were further crossed with wild-type C57BL/6 mice for at least three generations. Mice were genotyped by PCR analysis followed by sequencing and the resulted *Usp13*^+/m^ mice were crossed to generate *Usp13*^+/+^ and *Usp13*^m/m^ mice. Age- and sex-matched *Usp13*^+/+^ and *Usp13*^m/m^ littermates were blindingly randamonized into groups for animal studies. All mice were housed in the specific pathogen-free animal facility at Wuhan University and all animal experiments were in accordance with protocols approved by the Institutional Animal Care and Use Committee of Wuhan University.

### Co-immunoprecipitation and immunoblot assays

These experiments were performed as previously described^36^,^37^. Cells were collected and lysed for 15 min with 400 μl lysis buffer (20 mM Tris·HCl, pH 7.4–7.5, 150 mM NaCl, 1 mM EDTA, 1% Nonidet P-40) containing inhibitors for protease and phosphotases (Biotool). Cell lysates (300 μl) were incubated with a control IgG or specific antibodies and protein G agarose for 2–4 h. The immunoprecipitates were washed for three times by 1 ml prelysis buffer and subject to immunoblot analysis. The rest of lysates (100 μl) were subject to immunoblot analysis to detect the expression of target proteins.

### Deubiquitination assays

Cells were lysed in regular lysis buffer (100 μl) and the cell lysates were denatured at 95 °C for 5 min in the presence of 1% SDS. A portion of cell lysates (20 μl) were saved for immunoblot analysis to detect the expression of target proteins. The rest of cell lysates (80 μl) were diluted with 1 ml lysis buffer and immunoprecipitated (Denature-IP) with specific antibodies. The immunoprecipitates were washed by three times and subject to immunoblot analysis. Alternatively, cell lysates were pulled down by TUBE (LifeSensors, 50 μg ml^−1^) in the presence of 20 mM NEM followed by immunoblot analysis. For *in vitro* deubiquitination assays, denature-IP was performed to obtain ubiquitin-modified STING from HEK293 cells cotransfected with FLAG-tagged STING and HA-Ubiquitin. The immunoprecipitates were eluted by the FLAG peptide (0.5 mg ml^−1^, 60 μl). USP13 or USP13(AE) protein was obtained by an *in vitro* transcription and translation kit (Promega). The ubiquitin-modified FLAG-STING was incubated with USP13 or USP13(AE) at 37 °C for 2 h followed overnight incubation at 16 °C in the presence of ATP (1 μM). The mixtures were analysed by immunoblot with the indicated antibodies.

### Transfection and reporter gene assays

HEK293 cells were transiently transfected with firefly luciferase reporter (100 ng) and TK-Renilla luciferase reporter (20 ng) and indicated plasmids or empty vector (100 ng) using standard calcium phosphate precipitation. After 24 h, luciferase assays were performed with a dual-specific luciferase reporter kit (Promega). The activity of firefly luciferase was normalized by that of Renilla luciferase to obtain relative luciferase activity.

### Quantitative real-time PCR and ELISA

Total RNA was isolated from cells or tissues with TRIzol reagent (Life Technologies) and then was reserve-transcribed with All-in-One cDNA Synthesis SuperMix (Biotool). The cDNA was amplified by a fast two-step amplification program using 2 × SYBR Green Fast qPCR Master Mix and specific primers which were previously described. Data were normalized to the expression of the gene encoding β-actin. The IFN-α (PBL), IFN-β, IL-6 and CCl5 (Biolegend) protein in the sera or cell supernatants were determined by ELISA kits from the indicated manufacturers.

### Cell culture

Bone marrow cells were isolated from femurs of *Usp13*^+/+^ and *Usp13*^m/m^ mice. Primary MEFs were prepared from E14.5 embryos. The cells were cultured in DMEM containing 20% (vol/vol) FBS, 1% streptomycin-penicillin and 10 μM β-mercaptoethanol. GM-CSF, M-CSF and Flt3L (20 ng ml^−1^) were added to the bone marrow culture for differentiation of BMDCs, BMDMs and pDCs, respectively. CD11c^+^ BMDCs were selected by the CD11c magnetic beads (Stem Cell) for subsequent treatment. THP-1, HEK293 and HeLa cells were from the American Type Culture Collection, authenticated by STR locus analysis and tested for mycoplasma contamination^13^,^26^,^36^,^38^.

### Antibodies and reagents

Mouse control IgG (Santa Cruz Biotechnology, sc-2025) and rabbit control IgG (Millipore, 12–370), HRP-conjugated goat-anti mouse or rabbit IgG (Thermo Scientific, PA1-86717 and SA1-9510)(1:3,000), mouse anti-GFP (Sungene Biotech, KM8009)(1:1,000), mouse anti-FLAG (KM8002)(1:2,000), mouse anti-β-Actin (KM9001)(1:1,000), mouse anti-HA (COVANCE, MMS-101 R)(1:2,000), anti-pIκBα (9246L)(1:1,000), anti-Ubiquitin (sc-8017)(1:1,000), anti-IRF3 (sc-9082)(1:500), anti-IκBα (sc-371)(1:500), anti-p-IRF3 (4947 S)(1:1,000), anti-USP13 (abcam, GR56969-12)(1:500), anti-TBK1 (GR96328-11)(1:1,000) and anti-STING (13647 S)(1:1,000) were purchased from the indicated manufactures. Poly(I:C), ISD45, DNA90 and HSV120 were previously described^36^,^37^,^38^,^39^. ISD45: 5′-TACAGATCTACTAGTGATCTATGACTGATCTGTACATGATCTACA-3′; DNA90: 5′-TACAGATCTACTAGTGATCTATGACTGATCTGTACATGATCTACATACAGATCTACTAGTGATCTATGACTGATCTGTACATGATCTACA-3′; HSV120: 5′-AGACGGTATATTTTTGCGTTATCACTGTCCCGGATTGGACACGGTCTTGTGGGATAGGCATGCCCAGAAGGCATATTGGGTTAACCCCTTTTTATTTGTGGCGGGTTTTTTGGAGGACTT-3′.

### Viral infection

Cells were seeded into 24-well plates (2 × 10^5^ cells per well) or six-well plates (10^6^–10^7^ cells per well). After 24 h, cells were infected with HSV-1-GFP or HSV-1 for the indicated time points. The cells were collected for qPCR or immunoblot assays. For mice infection, eight to ten-week old and sex-matched *Usp13*^+/+^ and *Usp13*^m/m^ littermates were injected i.v. with HSV-1 (2.5 × 10^6^ PFU per mouse) and the survival of animals was monitored every day. The sera or lungs from mice infected with HSV-1 were collected for ELISA, qPCR or histological analysis at 12 or 24 h after infection, respectively. For viral replication assays, viruses were removed at 1 h after infection and cells were washed with 1 ml prewarmed PBS twice followed by culture with full medium for the indicated time points.

### Immunohistochemistry analysis

These experiments were performed as described previously^36^,^37^. Lungs from mice were fixed in formalin and embedded into paraffin blocks. The paraffin blocks were sectioned (5 μm) for H&E staining. The immunohistochemistry analysis was performed on the 5-μm sections. The sections were placed on polylysinecoated slides, deparaffinized in xylene, rehydrated through graded ethanol, quenched for endogenous peroxidase activity in 3% hydrogen peroxide, and processed for antigen retrieval by microwave heating for 7 min in 10 mM citrate buffer (pH 6.0). The anti-Ly6C antibody (M100L4; Tianjin Sungene Biotech) was diluted 1:100 in phosphate buffer saline (PBS) containing 1% BSA and incubated at room temperature for over 6 h. Immunostaining was performed using the Maixin_Bio Detection kit peroxidase/diaminobenzidine (DAB) rabbit/mouse (Kit-9710, DAB-0031; Maixin_Bio), which resulted in a brown-colored precipitate at the antigen site. Subsequently, sections were counterstained with hematoxylin (Zymed Laboratories) for 5 min and coverslipped. Pictures were acquired using a HistoFAXS system.

### Constructs

Various reporter plasmids and mammalian expression plasmids for STING, STING truncations, Rag9-GFP, MAVS, TBK1, IRF3, RIG-I CARD, ubiquitin and ubiquitin mutants were previously described^37^,^38^. Mammalian expression plasmids for USP13, USP13 mutants and truncations were constructed by standard molecular biology techniques. Oligo DNAs targeting USP13 were synthesized, annealed and inserted into the pLenti-GFP vector. The sequences of USP13 shRNA are as following: #1: 5′-GTGATTGAGATGGAGAATA-3′; #2: 5′-GGGAACATGTTGAAAGACA-3′. Plasmids encoding cGAS and blue fluorescence protein (BFP) were kindly provided by Dr Zhijian Chen (UT Southwestern) and Dr Jun Chu (Shenzhen Institutes of Advanced Technology), respectively.

### Lentivirus-mediated gene transfer

HEK293 cells (China Center for Type Culture Collection, Wuhan University, tested for mycoplasma negative) were transfected with phage-USP13-FLAG, phage-USP13(AE)-FLAG or the empty vector along with the packaging vectors pSPAX2 and pMD2G. The shRNA lentivurses were packaged by cotransfection of the control or USP13 shRNA vector together with pSPAX2 and pMD2G into HEK293 cells. Eight hours later, the medium was changed with fresh full medium (10% FBS, 1% streptomycin-penicillin and 10 μM β-mercaptoethanol). Forty hours later, the supernatants were collected to infect MEFs, BMDCs or THP-1 cells.

### siRNA

The siRNAs targeting USP13 were synthesized and transfected into cells by lipofectatmine 2,000 (Life Technologies) followed by qPCR or Immunoblot analysis. The siRNA sequences used in this study are as following: siUSP13#1: 5′-GCUCUGUCCUGUGUGGAAATT-3′; siUSP13#2: 5′-GCACGAAACUGAAGCCAAUTT-3′; siAMFR#1: 5′-GCAUGCACACCUUGGCUUUTT-3′; siAMFR#2: 5′-GGUUAUCUAUGGCUAGCUUTT-3′; siAMFR#3: 5′-GCACACUACCAACAUUCUUTT-3′; N.C.: 5′-UUCUCCGAACGUGUCACGUTT-3′.

### Plaque assay

The supernatants of BMDCs or MEFs cultures and the homogenates of brains from infected mice (or the serial dilutions) were used to infect monolayers of Vero cells. One hour later, the supernatants or homogenates were removed and the infected Vero cells were washed with pre-warmed PBS twice followed by incubation with DMEM containing 2% methylcellulose for 48 h. The cells were fixed with 4% paraformaldehyde for 15 min and stained with 1% crystal violet for 30 min before counting the plaques.

### Immunofluorescence and confocal microscopy analysis

HeLa cells cultured on coverslips were fixed in 4% paraformaldehyde for 10 min and washed with PBS for three times. After that, cells were fixed with 0.1% Triton X-100 on ice for 5 min, washed in PBS and blocked in 1% BSA for 20 min. The coverslips were incubated with 1% BSA containing primary antibodies for 1 h followed by PBS wash for three times. The cells were further stained with Alexa Fluor 488- or 594- conjugated secondary antibodies. Images were acquired on an Olympus FV1000 fluorescence microscope.

### Statistical analysis

Differences between experimental and control groups were determined by Student's *t* test (where two groups of data were compared) or by two-way ANOVA analysis (where more than two groups of data were compared). *P* values <0.05 were considered statistically significant. For animal survival analysis, the Kaplan–Meier method was adopted to generate graphs, and the survival curves were analysed with log-rank analysis.

### Data availability

The data that support the findings of this study are available from the corresponding author on request.

## Additional information

**How to cite this article:** Sun, H. *et al*. USP13 negatively regulates antiviral responses by deubiquitinating STING. *Nat. Commun.* **8**, 15534 doi: 10.1038/ncomms15534 (2017).

**Publisher's note**: Springer Nature remains neutral with regard to jurisdictional claims in published maps and institutional affiliations.

## Supplementary Material

Supplementary Information Supplementary Figures

## References

[b1] Takeuchi O. & Akira S. Pattern recognition receptors and inflammation. Cell 140, 805–820 (2010). 20303872 10.1016/j.cell.2010.01.022

[b2] Roers A., Hiller B. & Hornung V. Recognition of endogenous nucleic acids by the innate immune system. Immunity 44, 739–754 (2016). 27096317 10.1016/j.immuni.2016.04.002

[b3] Wu J. & Chen Z. J. Innate immune sensing and signaling of cytosolic nucleic acids. Annu. Rev. Immunol. 32, 461–488 (2014). 24655297 10.1146/annurev-immunol-032713-120156

[b4] Takaoka A. . DAI (DLM-1/ZBP1) is a cytosolic DNA sensor and an activator of innate immune response. Nature 448, 501–505 (2007). 17618271 10.1038/nature06013

[b5] Chiu Y. H., Macmillan J. B. & Chen Z. J. RNA polymerase III detects cytosolic DNA and induces type I interferons through the RIG-I pathway. Cell 138, 576–591 (2009). 19631370 10.1016/j.cell.2009.06.015 PMC2747301

[b6] Ablasser A. . RIG-I-dependent sensing of poly(dA:dT) through the induction of an RNA polymerase III-transcribed RNA intermediate. Nat. Immunol. 10, 1065–1072 (2009). 19609254 10.1038/ni.1779 PMC3878616

[b7] Unterholzner L. . IFI16 is an innate immune sensor for intracellular DNA. Nat. Immunol. 11, 997–1004 (2010). 20890285 10.1038/ni.1932 PMC3142795

[b8] Zhang Z. . The helicase DDX41 senses intracellular DNA mediated by the adaptor STING in dendritic cells. Nat. Immunol. 12, 959–965 (2011). 21892174 10.1038/ni.2091 PMC3671854

[b9] Li Y. . LSm14A is a processing body-associated sensor of viral nucleic acids that initiates cellular antiviral response in the early phase of viral infection. Proc. Natl Acad. Sci. USA 109, 11770–11775 (2012). 22745163 10.1073/pnas.1203405109 PMC3406844

[b10] Sun L., Wu J., Du F., Chen X. & Chen Z. J. Cyclic GMP-AMP synthase is a cytosolic DNA sensor that activates the type I interferon pathway. Science 339, 786–791 (2013). 23258413 10.1126/science.1232458 PMC3863629

[b11] Gao P. . Cyclic [G(2′,5′)pA(3′,5′)p] is the metazoan second messenger produced by DNA-activated cyclic GMP-AMP synthase. Cell 153, 1094–1107 (2013). 23647843 10.1016/j.cell.2013.04.046 PMC4382009

[b12] Ablasser A. . cGAS produces a 2′-5′-linked cyclic dinucleotide second messenger that activates STING. Nature 498, 380–384 (2013). 23722158 10.1038/nature12306 PMC4143541

[b13] Zhong B. . The adaptor protein MITA links virus-sensing receptors to IRF3 transcription factor activation. Immunity 29, 538–550 (2008). 18818105 10.1016/j.immuni.2008.09.003

[b14] Ishikawa H. & Barber G. N. STING is an endoplasmic reticulum adaptor that facilitates innate immune signalling. Nature 455, 674–678 (2008). 18724357 10.1038/nature07317 PMC2804933

[b15] Sun W. . ERIS, an endoplasmic reticulum IFN stimulator, activates innate immune signaling through dimerization. Proc. Natl Acad. Sci. USA 106, 8653–8658 (2009). 19433799 10.1073/pnas.0900850106 PMC2689030

[b16] Jin L. . MPYS is required for IFN response factor 3 activation and type I IFN production in the response of cultured phagocytes to bacterial second messengers cyclic-di-AMP and cyclic-di-GMP. J. Immunol. 187, 2595–2601 (2011). 21813776 10.4049/jimmunol.1100088 PMC3159690

[b17] Wu J. . Cyclic GMP-AMP is an endogenous second messenger in innate immune signaling by cytosolic DNA. Science 339, 826–830 (2013). 23258412 10.1126/science.1229963 PMC3855410

[b18] Cai X., Chiu Y. H. & Chen Z. J. The cGAS-cGAMP-STING pathway of cytosolic DNA sensing and signaling. Mol. Cell 54, 289–296 (2014). 24766893 10.1016/j.molcel.2014.03.040

[b19] You F. . ELF4 is critical for induction of type I interferon and the host antiviral response. Nat. Immunol. 14, 1237–1246 (2013). 24185615 10.1038/ni.2756 PMC3939855

[b20] Holm C. K. . Influenza A virus targets a cGAS-independent STING pathway that controls enveloped RNA viruses. Nat. Commun. 7, 10680 (2016). 26893169 10.1038/ncomms10680 PMC4762884

[b21] Ahn J., Gutman D., Saijo S. & Barber G. N. STING manifests self DNA-dependent inflammatory disease. Proc. Natl Acad. Sci. USA 109, 19386–19391 (2012). 23132945 10.1073/pnas.1215006109 PMC3511090

[b22] Liu Y. . Activated STING in a vascular and pulmonary syndrome. N. Engl. J. Med. 371, 507–518 (2014). 25029335 10.1056/NEJMoa1312625 PMC4174543

[b23] Tsuchida T. . The ubiquitin ligase TRIM56 regulates innate immune responses to intracellular double-stranded DNA. Immunity 33, 765–776 (2010). 21074459 10.1016/j.immuni.2010.10.013

[b24] Zhang J., Hu M. M., Wang Y. Y. & Shu H. B. TRIM32 protein modulates type I interferon induction and cellular antiviral response by targeting MITA/STING protein for K63-linked ubiquitination. J. Biol. Chem. 287, 28646–28655 (2012). 22745133 10.1074/jbc.M112.362608 PMC3436586

[b25] Zhong B. . The ubiquitin ligase RNF5 regulates antiviral responses by mediating degradation of the adaptor protein MITA. Immunity 30, 397–407 (2009). 19285439 10.1016/j.immuni.2009.01.008

[b26] Qin Y. . RNF26 temporally regulates virus-triggered type I interferon induction by two distinct mechanisms. PLoS Pathogens 10, e1004358 (2014). 25254379 10.1371/journal.ppat.1004358 PMC4177927

[b27] Wang Q. . The E3 ubiquitin ligase AMFR and INSIG1 bridge the activation of TBK1 kinase by modifying the adaptor STING. Immunity 41, 919–933 (2014). 25526307 10.1016/j.immuni.2014.11.011

[b28] Liu J. . Beclin1 controls the levels of p53 by regulating the deubiquitination activity of USP10 and USP13. Cell 147, 223–234 (2011). 21962518 10.1016/j.cell.2011.08.037 PMC3441147

[b29] Zhang J. . Deubiquitylation and stabilization of PTEN by USP13. Nat. Cell Biol. 15, 1486–1494 (2013). 24270891 10.1038/ncb2874 PMC3951854

[b30] Zhao X., Fiske B., Kawakami A., Li J. & Fisher D. E. Regulation of MITF stability by the USP13 deubiquitinase. Nat. Commun. 2, 414 (2011). 21811243 10.1038/ncomms1421

[b31] Yeh H. M. . Ubiquitin-specific protease 13 regulates IFN signaling by stabilizing STAT1. J. Immunol. 191, 3328–3336 (2013). 23940278 10.4049/jimmunol.1300225

[b32] Liu Y. . USP13 antagonizes gp78 to maintain functionality of a chaperone in ER-associated degradation. eLife 3, e01369 (2014). 24424410 10.7554/eLife.01369 PMC3889402

[b33] Ran Y., Shu H. B. & Wang Y. Y. MITA/STING: a central and multifaceted mediator in innate immune response. Cytokine Growth Factor Rev. 25, 631–639 (2014). 24929887 10.1016/j.cytogfr.2014.05.003 PMC7108248

[b34] Ishikawa H., Ma Z. & Barber G. N. STING regulates intracellular DNA-mediated, type I interferon-dependent innate immunity. Nature 461, 788–792 (2009). 19776740 10.1038/nature08476 PMC4664154

[b35] Sharma S. . Suppression of systemic autoimmunity by the innate immune adaptor STING. Proc. Natl Acad. Sci. USA 112, E710–E717 (2015). 25646421 10.1073/pnas.1420217112 PMC4343138

[b36] Zhang M. . USP18 recruits USP20 to promote innate antiviral response through deubiquitinating STING/MITA. Cell Res. 26, 1302–1319 (2016). 27801882 10.1038/cr.2016.125 PMC5143414

[b37] Lin D. . Induction of USP25 by viral infection promotes innate antiviral responses by mediating the stabilization of TRAF3 and TRAF6. Proc. Natl Acad. Sci. USA 112, 11324–11329 (2015). 26305951 10.1073/pnas.1509968112 PMC4568686

[b38] Zhou Q. . The ER-associated protein ZDHHC1 is a positive regulator of DNA virus-triggered, MITA/STING-dependent innate immune signaling. Cell Host Microbe 16, 450–461 (2014). 25299331 10.1016/j.chom.2014.09.006

[b39] Luo W. W. . iRhom2 is essential for innate immunity to DNA viruses by mediating trafficking and stability of the adaptor STING. Nat. Immunol. 17, 1057–1066 (2016). 27428826 10.1038/ni.3510

