# Supplementary material for: USP13 negatively regulates antiviral responses by deubiquitinating STING
Source: Nat Commun. 2017 May 23;8:15534. doi: 10.1038/ncomms15534 (PMC5457515; doi:10.1038/ncomms15534)

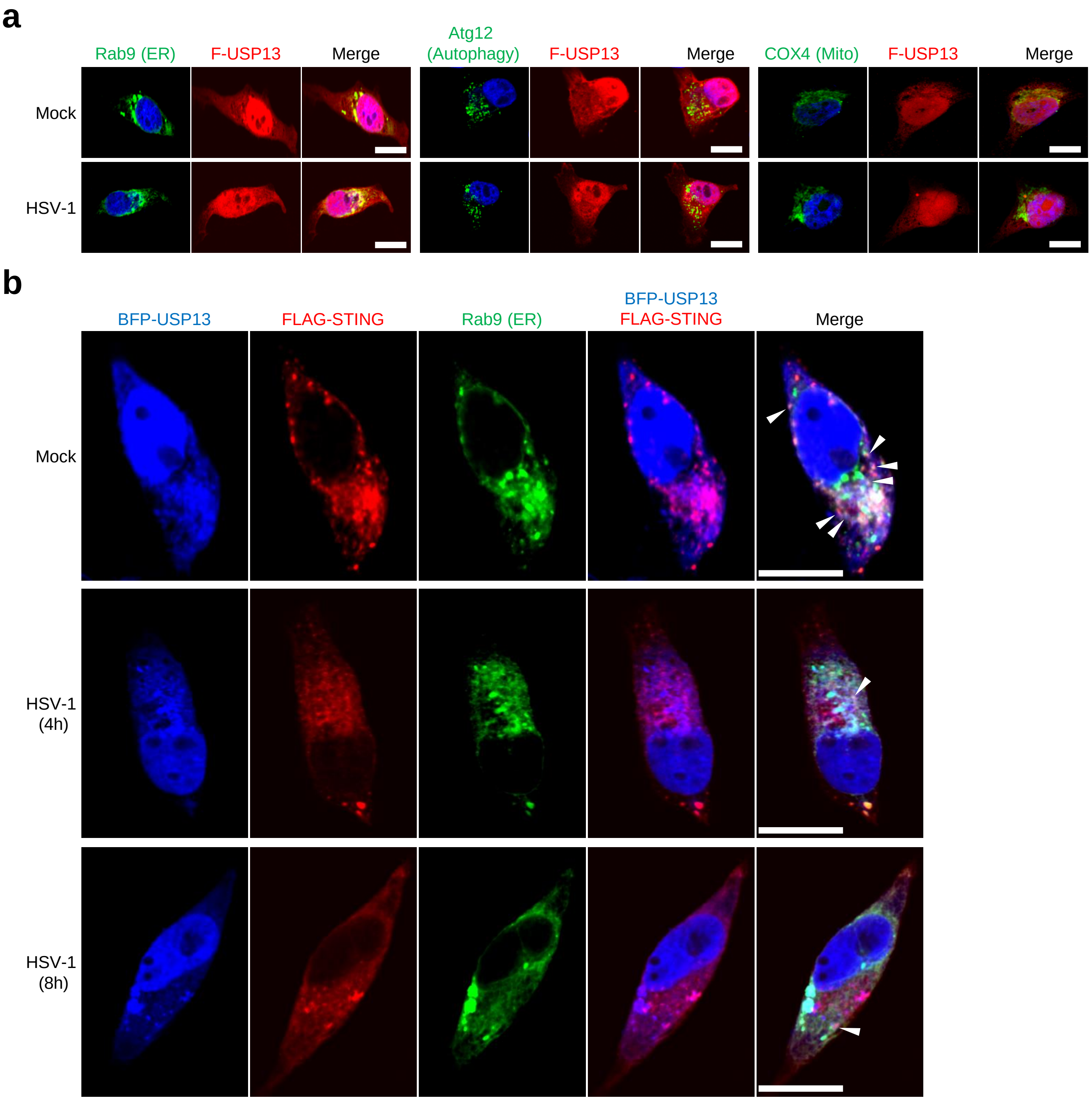

Supplementary Figure 1 USP13 and STING colocalize at ER.

(**a**) Immunofluorescence staining (with anti-Atg12, anti-COX4 or anti-FLAG) and confocal microscopy analysis of HeLa cells transfected with Rab9 (an ER marker) and FLAG-USP13 for 20 hours followed by mock infection or HSV-1 infection for 8 hours.

(**b**) Immunofluorescence and confocal microscopy analysis of HeLa cells transfected with Rab9, BFP-USP13 and FLAG-STING for 20 hours followed by HSV-1 infection for 0-8 hours. Arrow heads indicate colocalization of STING and USP13 at ER.

Scale bars represent 20 μm. Data are representative of three independent experiments.

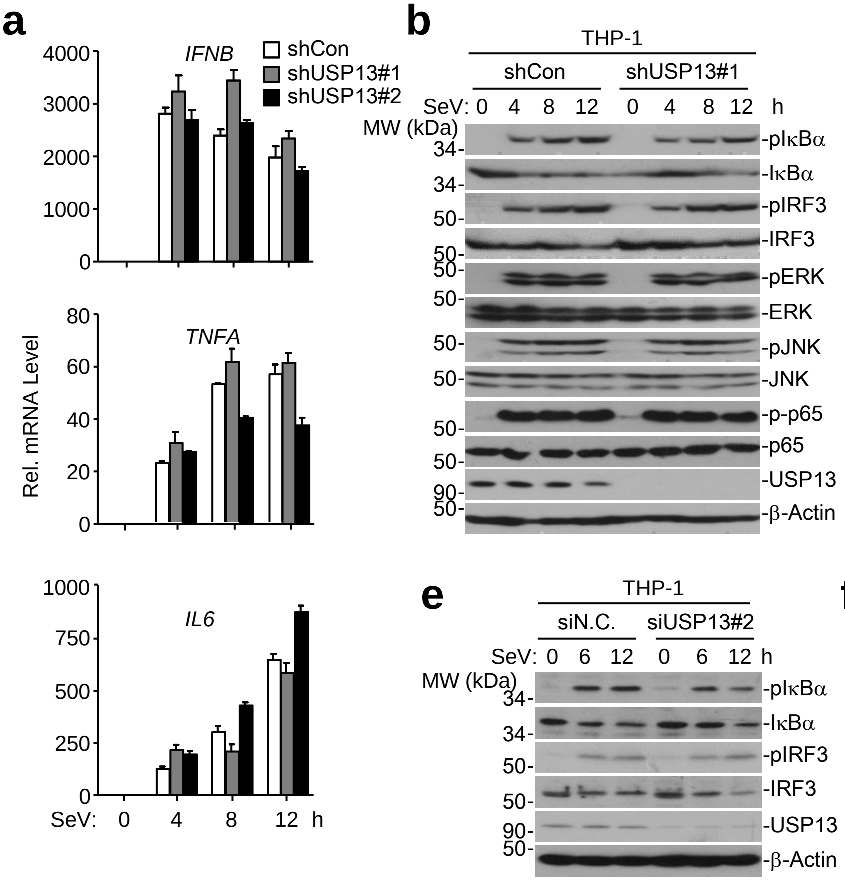

Supplementary Figure 2 Knockdown of USP13 did not affect SeV-triggered signaling.

(**a**) qRT-PCR analysis of *IFNB*, *TNFA*, and *IL6* in THP-1 cells stably transfected with control shRNA, shUSP13#1 or shUSP13#2, then infected with HSV-1 for 0-24 hours.

(**b**) Immunoblot analysis of phosphorylated and total IRF3, IκBα, ERK, p38, and JNK and β-Actin in THP-1 cells stably transfected with control shRNA or shUSP13#1, then infected with HSV-1 for 0-12 hours.

(**c**) Immunoblot analysis (with anti-USP13 or anti-β-Actin) of THP-1 cells transfected siRNAs targeting USP13 (#1, #2, #3) or non-targeting control siRNA(N.C.) for 36 hours.

(**d**) qRT-PCR analysis of *IFNB* and *TNFA* in THP-1 cells transfected with siUSP13#2 or N.C. for 36 hours, then infected with HSV-1 or SeV for 0-8 hours.

(**e-f**) Immunoblot analysis of phosphorylation of IRF3 and IκBα, total IRF3 and IκBα, USP13 and β-Actin in THP-1 cells transfected with siUSP13#2 or N.C. for 36 hours, then infected with SeV (e) or HSV-1 (f) for 0-8 hours.

*P < 0.05; ***P <0.001 (analysis of two-way ANOVA followed by Bonferroni post-test). Data are representative of three independent experiments (mean ± S.D. in **a** and **d**).

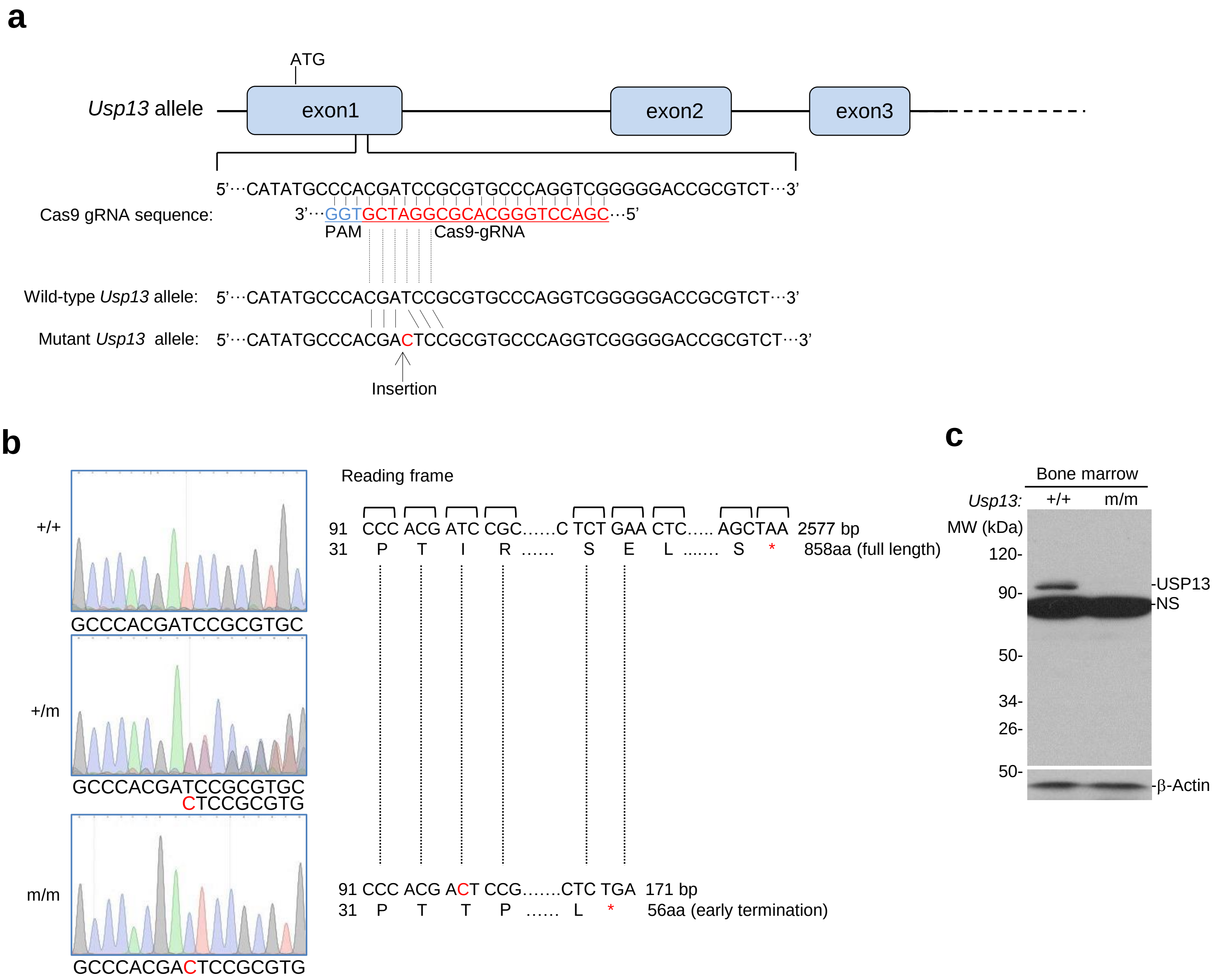

Supplementary Figure 3 Generation of *Usp13*$^{m/m}$ mice.
(**a**) A scheme for CRIPSR/Cas9-mediated genome editing of the *Usp13* gene locus.
(**b**) Gene sequence and reading frame of *Usp13*$^{+/+}$, *Usp13*$^{+/m}$ and *Usp13*$^{m/m}$ mice.
(**c**) Immunoblot analysis of USP13 in bone marrow cells from *Usp13*$^{+/+}$ and *Usp13*$^{m/m}$ mice.
Data are representative of two (**c**) independent experiments.

a

Genotype	*Usp13*+/+	*Usp13*+/m	*Usp13*m/m
Number	121	258	110
Percentage	24.7%	52.8%	22.5%

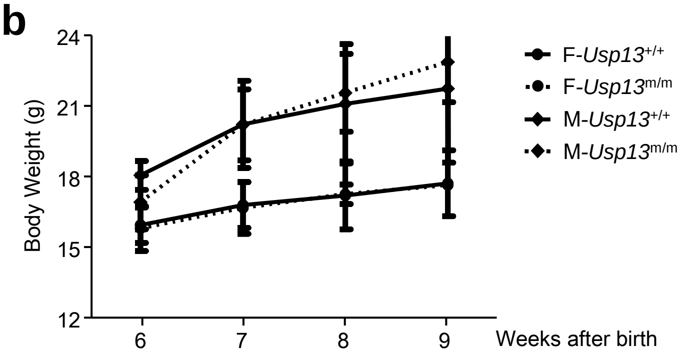

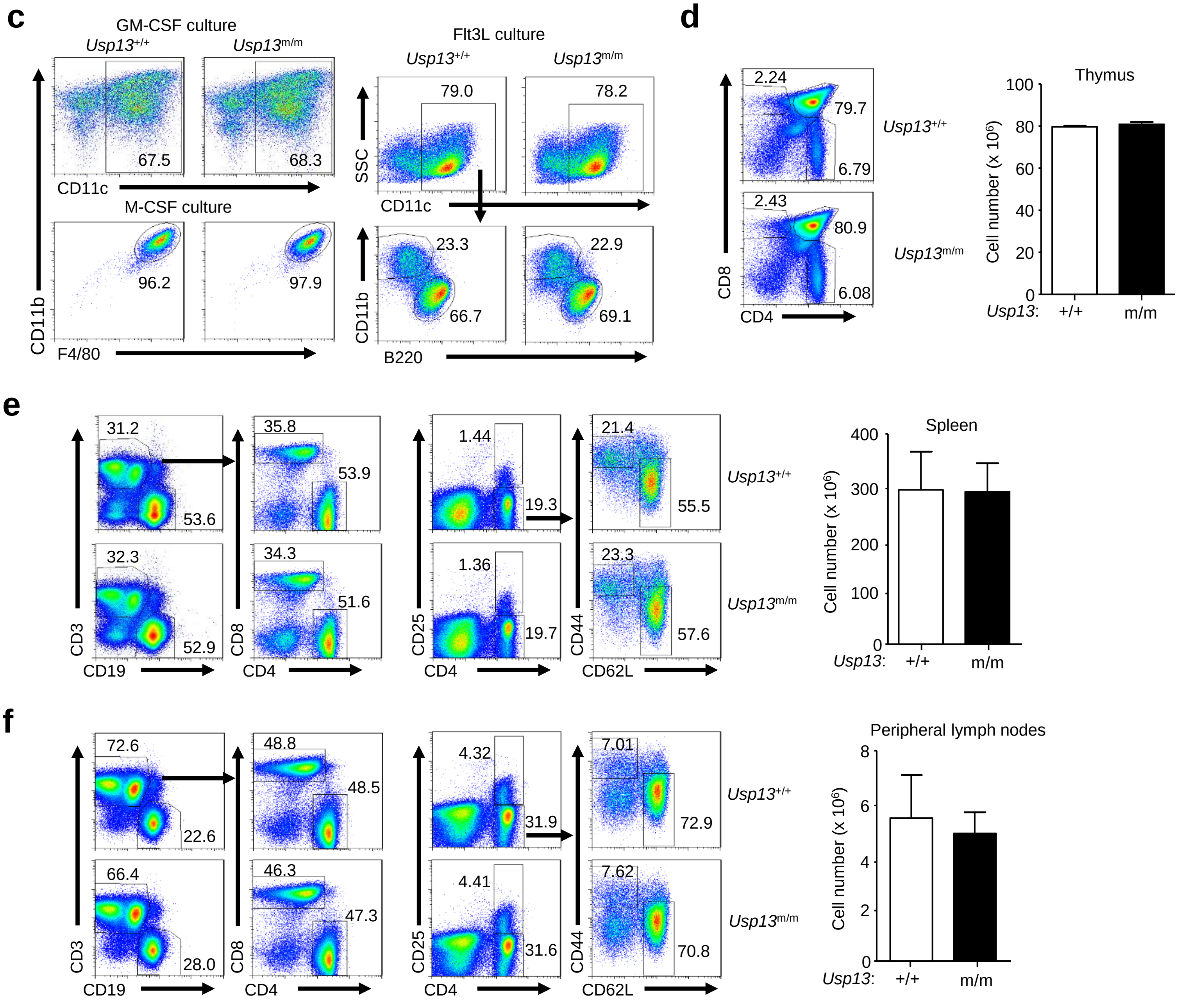

Supplementary Figure 4 USP13 deficiency did not alter the homeostasis of immune cells.
(**a**) Mice numbers and percentages of each genotype.
(**b**) Mice body weight of each genotype. F, Female; M, Male.
(**c**) Flow cytometry analysis of GM-CSF, M-CSF or Flt3L induced DCs, Macrophage cells or pDCs from *Usp13*+/+ and *Usp13*m/m mice.
(**d-f**) Flow cytometry analysis of immune cells and quantitative data in thymus (**e**), spleen (**f**) and peripheral lymph nodes from *Usp13*+/+ and *Usp13*m/m mice.
Data are representative of two (**c-f**) independent experiments (n=3) (mean ± S.D. in **b, d-f**).

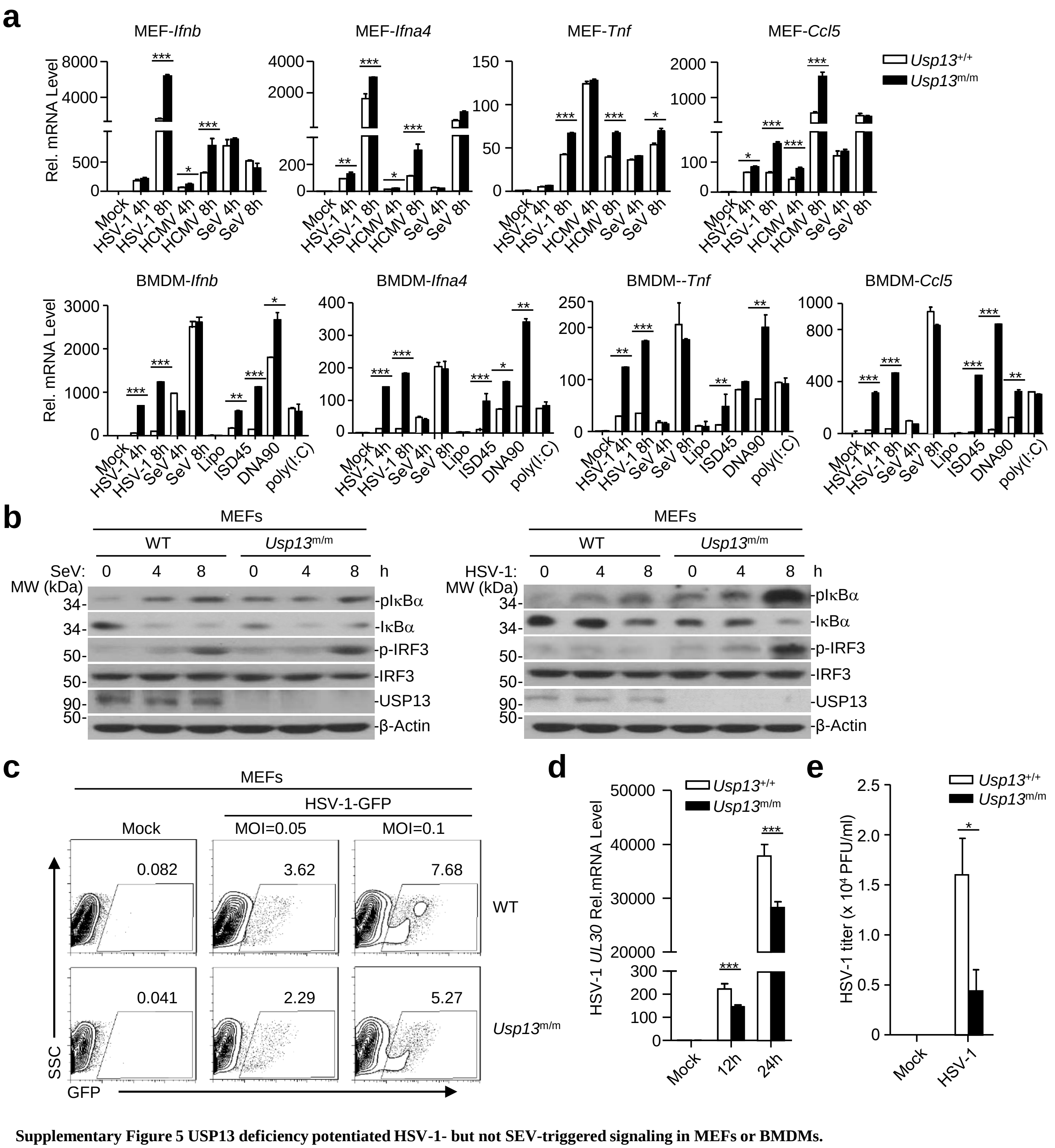

Supplementary Figure 5 USP13 deficiency potentiated HSV-1- but not SEV-triggered signaling in MEFs or BMDMs.

(**a**) qRT-PCR analysis of *Ifnb*, *Ifna4*, *Tnf* or *Ccl5* mRNA in *Usp13*$^{+/+}$ and *Usp13*$^{m/m}$ MEFs or BMDMs left uninfected (Mock) or infected with HSV-1, SeV, HCMV or EMCV for 0-8 hours, or mock transfected (Lipo) or transfected with ISD45, DNA90 or poly(I:C) for 6 hours.

(**b**) Immunoblot analysis of phosphorylation of IRF3 and IκBα, total IRF3 and IκBα, USP13 and β-Actin in *Usp13*$^{+/+}$ and *Usp13*$^{m/m}$ MEFs infected with SeV or HSV-1 for 0-8 hours.

(**c**) Flow cytometry analysis of *Usp13*$^{+/+}$ and *Usp13*$^{m/m}$ MEFs infected with HSV-1-GFP for 24 hours. Numbers adjacent to outlined areas indicate the percentages of GFP^{+} MEFs.

(**d**) qRT-PCR analysis of HSV-1-*UL30* mRNA in *Usp13*$^{+/+}$ and *Usp13*$^{m/m}$ MEFs infected with HSV-1 for 1 hour followed by twice PBS wash and subsequent incubation in full medium for 0-24 hours.

(**e**) Viral plaque assay of HSV-1 in *Usp13*$^{+/+}$ and *Usp13*$^{m/m}$ MEFs infected with HSV-1 for 1 hour followed by twice PBS wash and cultured in full medium for 32 hours.

P* < 0.05; ***P* < 0.01; ****P* <0.001 (analysis of two-way ANOVA followed by Bonferroni post-test). Data are representative of three (a, b**) or two (**c-e**) independent experiments (mean ± S.D. in **a, d-e**).

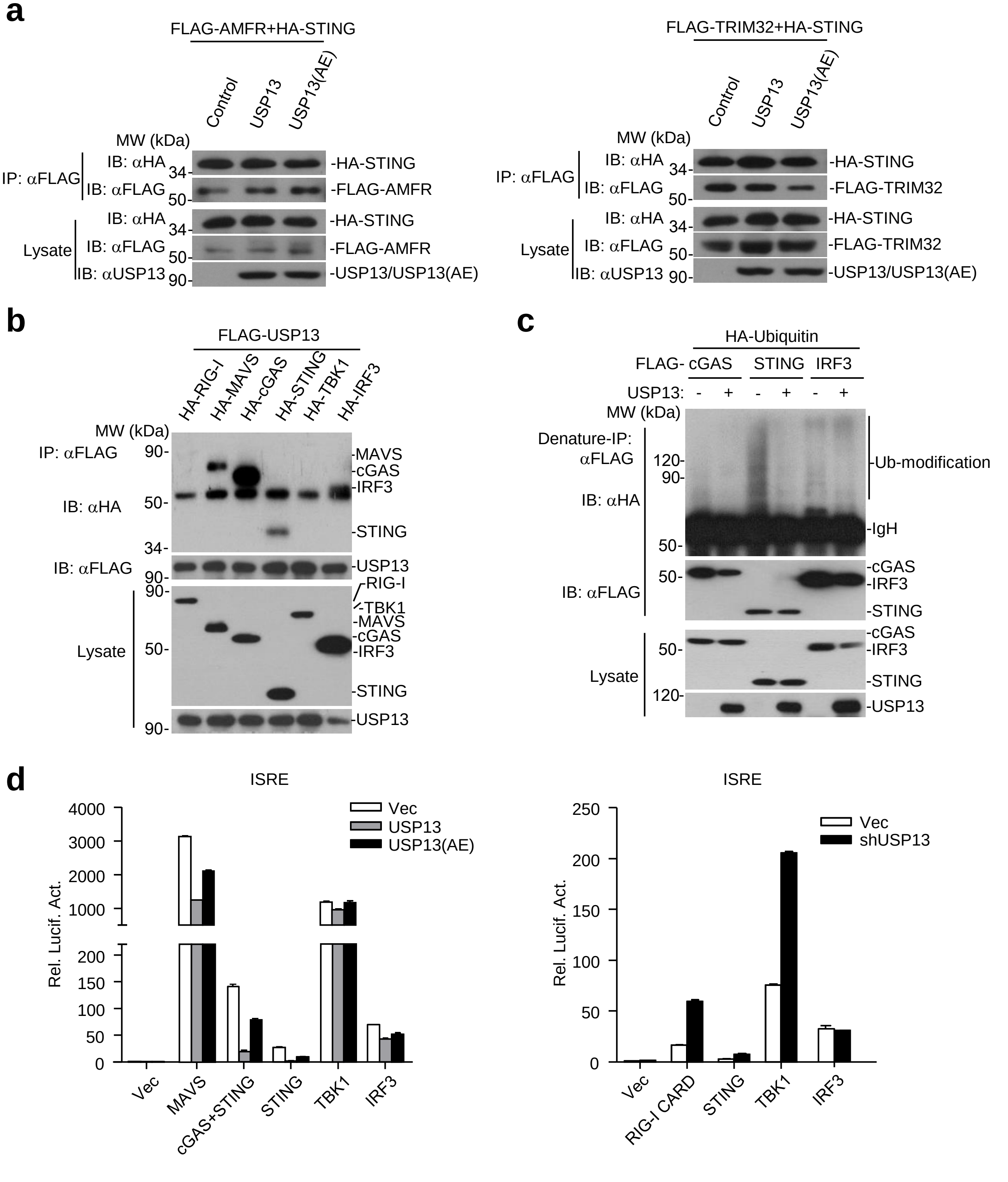

Supplementary Figure 6 USP13 functions at the level of STING.

(**a**) Immunoprecipitation (with anti-FLAG) and immunoblot analysis (with anti-FLAG, anti-HA or anti-USP13) of HEK293 cells transfected with plasmids encoding FLAG-tagged AMFR or TRIM32, HA-STING and USP13 or USP13 enzymatic inactive mutation for 24 hours.

(**b**) Immunoprecipitation (with anti-FLAG) and immunoblot analysis (with anti-FLAG or anti-HA) of HEK293 cells transfected with plasmids encoding FLAG-USP13 and HA-tagged RIG-I, MAVS, cGAS, STING, TBK1 or IRF3 for 24 hours.

(**c**) Denature-immunoprecipitation (with anti-FLAG) and immunoblot analysis (with anti-FLAG, anti-HA or anti-USP13) of HEK293 cells transfected with plasmids encoding HA-ubiquitin, FLAG-tagged cGAS, STING or IRF3, and either empty vector or USP13 for 24 hours.

(**d**) Luciferase assay analyzing ISRE promoter activity in HEK293 cells transfected with plasmids encoding MAVS, cGAS, STING, TBK1 or IRF3, an ISRE firefly luciferase reporter (ISRE-Luc) and the empty vector, USP13 or USP13(AE) for 24 hours (left graph). Luciferase assay analyzing ISRE promoter activity in HEK293 cells transfected with plasmids encoding RIG-I CARD, STING, TBK1 or IRF3, an ISRE firefly luciferase reporter (ISRE-Luc) and either the empty vector or shRNA targeting USP13 for 24 hours (right).

Data are representative of two independent experiments (mean ± S.D. in **d**).

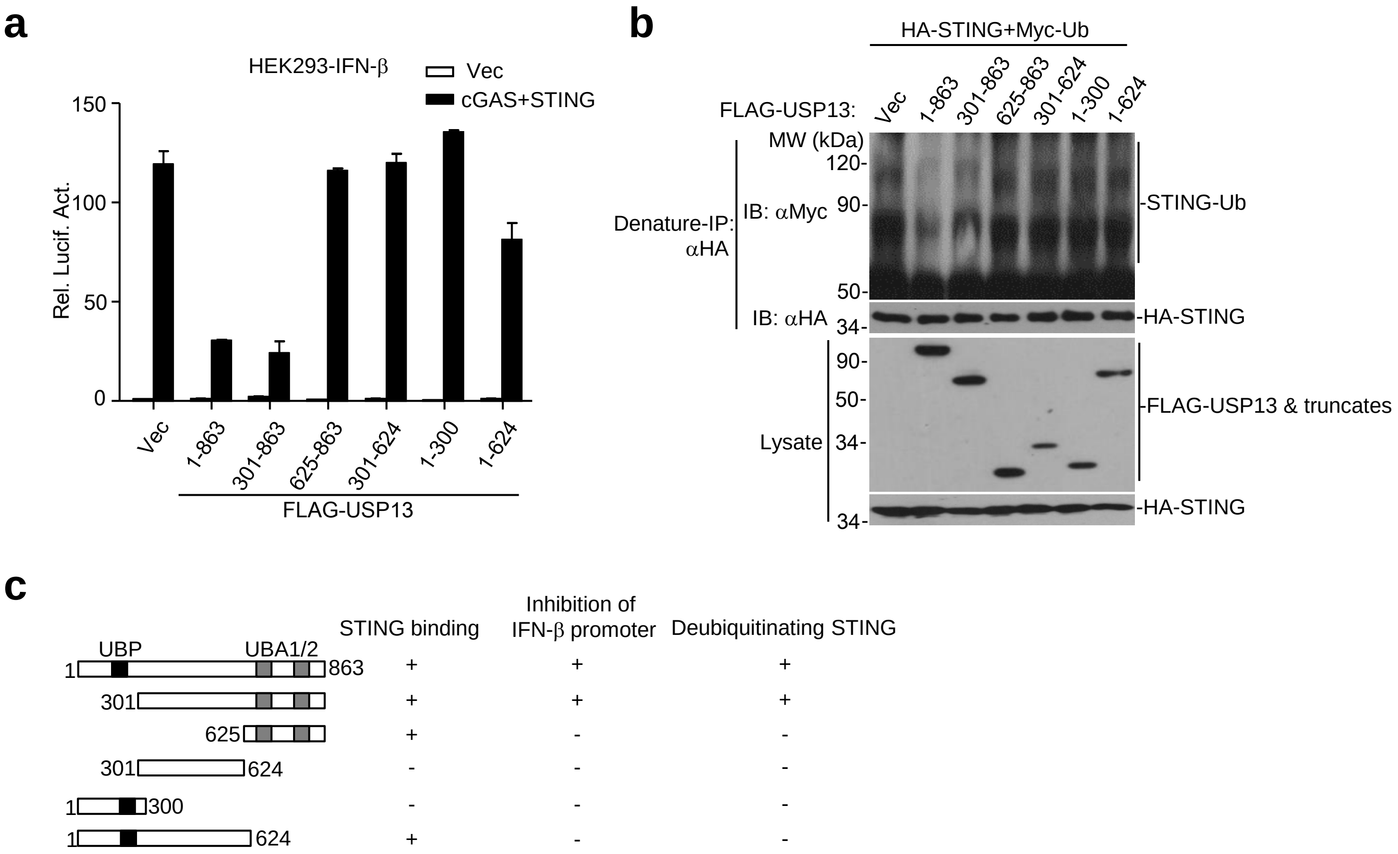

Supplementary Figure 7 The isopeptidase and the UBA domains of USP13 deubiquitinate STING.

(**a**) Reporter assays of HEK293 cells transfected with the indicated truncates of USP13 together with the IFN-β luciferase reporter for 24 hours.

(**b**) Denature-IP (with anti-HA) and immunoblot analysis (with anti-FLAG or anti-Myc) of HEK293 cells transfected with plasmids encoding Myc-Ubiquitin, HA-STING, and FLAG-tagged USP13 or USP13 truncates for 20 hours.

(**c**) Schematic structures showing USP13 truncates interacting with STING, inhibiting cGAS and STING-mediated activation of IFN-b promoter and deubiquitinating STING. Data are representative of two independent experiments (mean ± S.D. in **a**).

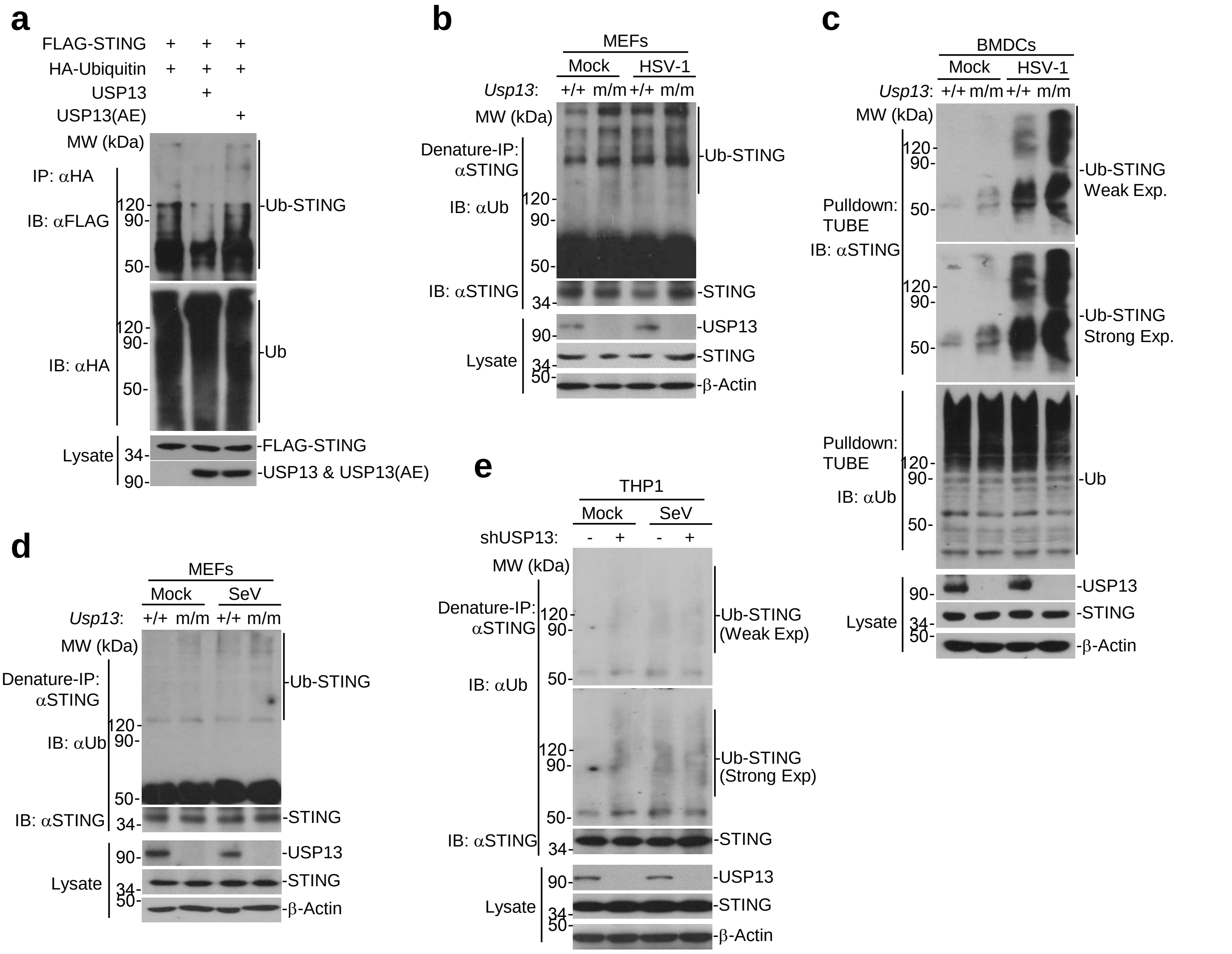

Supplementary Figure 8 USP13 did not regulate SeV-induced ubiquitination of STING.

(**a**) Immunoprecipitation (with anti-HA) and immunoblot analysis (with anti-FLAG, anti-HA, or anti-USP13) of HEK293 cells transfected with plasmids encoding FLAG-STING, HA-Ubiquitin and USP13 or USP13(AE) for 24 hours.

(**b**) Denature-immunoprecipitation (with anti-STING) and immunoblot analysis (with anti-Ub, anti-STING, anti-USP13 or anti-β-Actin) of *Usp13*$^{+/+}$ and *Usp13*$^{m/m}$ MEFs infected for 8h with HSV-1.

(**c**) Immunoprecipitation (with GST beads and TUBE) and immunoblot analysis (with anti-STING, anti-Ub, anti-USP13, anti-β-Actin) of *Usp13*$^{+/+}$ or *Usp13*$^{m/m}$ BMDCs infected with HSV-1 for 0-8 hours.

(**d-e**) Denature-immunoprecipitation (with anti-STING) and immunoblot analysis (with anti-Ub, anti-STING, anti-USP13 or anti-β-Actin) of *Usp13*$^{+/+}$ and *Usp13*$^{m/m}$ MEFs (**d**) or USP13-knockdown-THP-1cells (**e**) infected for 8h with SeV.

Data are representative of three independent experiments.

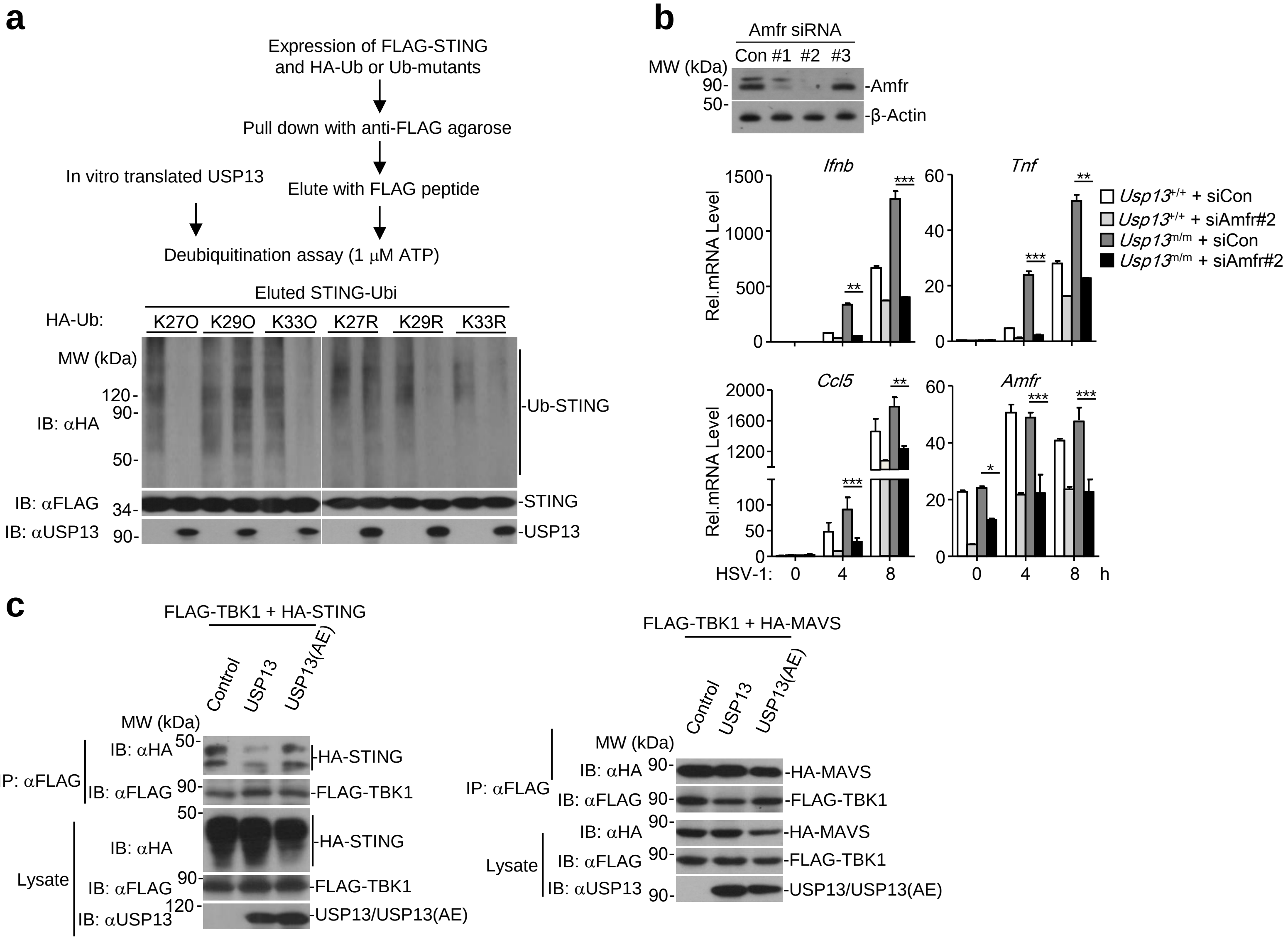

Supplementary Figure 9 USP13 deconjugates K37/K33-linked polyubiquitin chains from STING.

(**a**) In vitro deubiquitination of Ubiquitin-modified STING by USP13.

(**b**) Immunoblot (upper panels) and qRT-PCR (lower graphs) analysis of *Usp13*$^{+/+}$ and *Usp13*$^{m/m}$ MEFs that were transfected with control siRNA or siRNA targeting Amfr for 36 hours and then left untreated or infected with HSV-1 for 4-8 hours.

(**c**) Immunoprecipitation (with anti-FLAG) and immunoblot analysis (with anti-FLAG, anti-HA or anti-USP13) of HEK293 cells transfected with plasmids encoding FLAG-TBK1, HA-STING, HA-MAVS and the empty vector (Control), USP13 or USP13(AE) for 20 hours.

P* < 0.05; ***P* < 0.01; ****P* <0.001 (analysis of two-way ANOVA followed by Bonferroni post-test). Data are representative of three independent experiments (mean ± S.D. in **c).

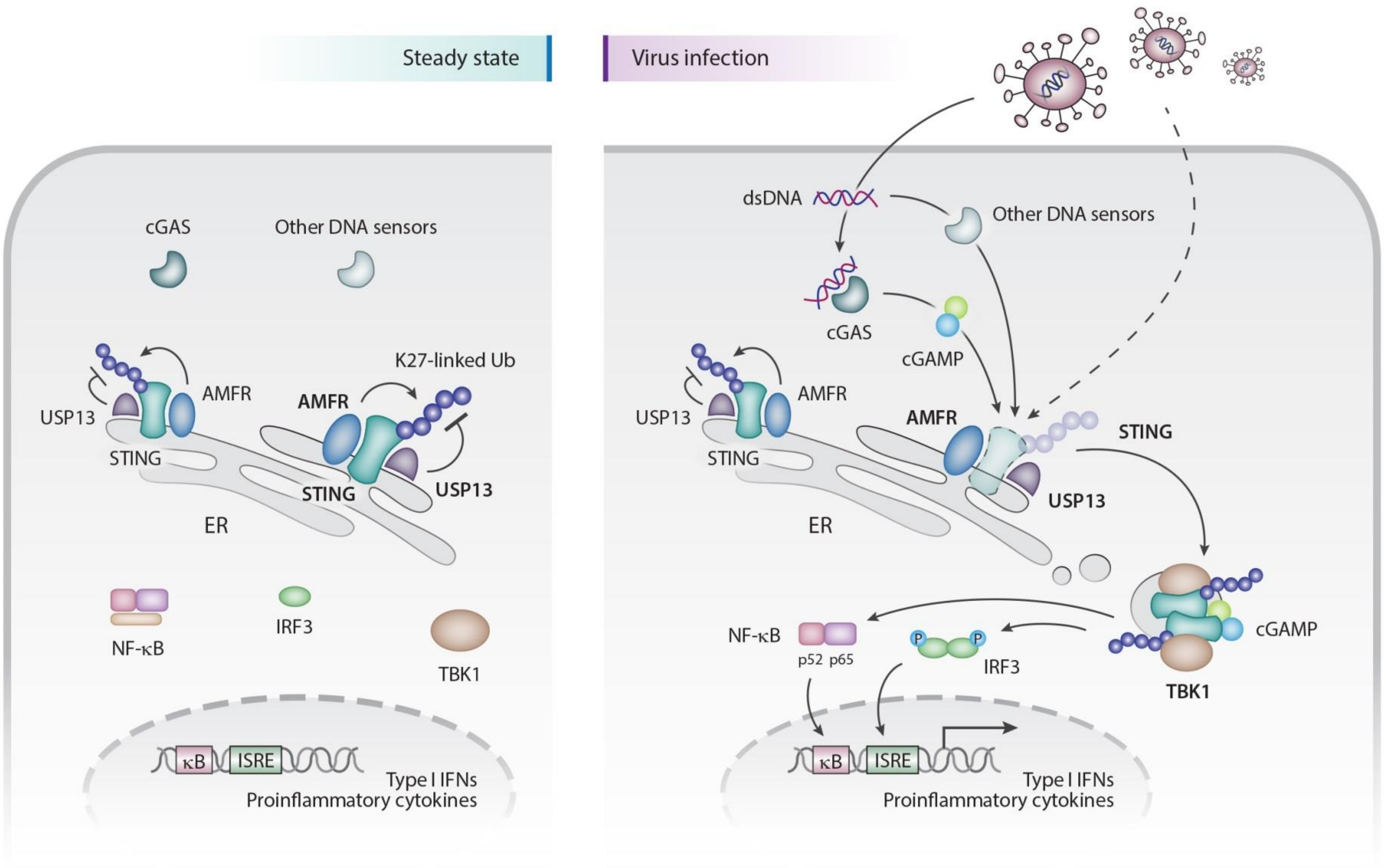

Supplementary Figure 10 A model on USP13-mediated regulation of antiviral responses.
In unstimulated cells, USP13 interacts with and catalyzes deubiquitination of STING which constitutively undergoes K27-linked ubiquitination mediated by AMFR/gp78, thereby restricting the basal immune signaling. Upon infection with DNA viruses, the majority of USP13-interacting STING is disassociated from USP13 and translocates to form puncta, thereby leading to increased ubiquitination of STING and boosted recruitment of TBK1. Meanwhile, a portion of USP13 still interacts with STING to prevent excessive immune and inflammatory responses.

Supplementary Figure 11 Original immunoblots for Figure 1

Figure 1

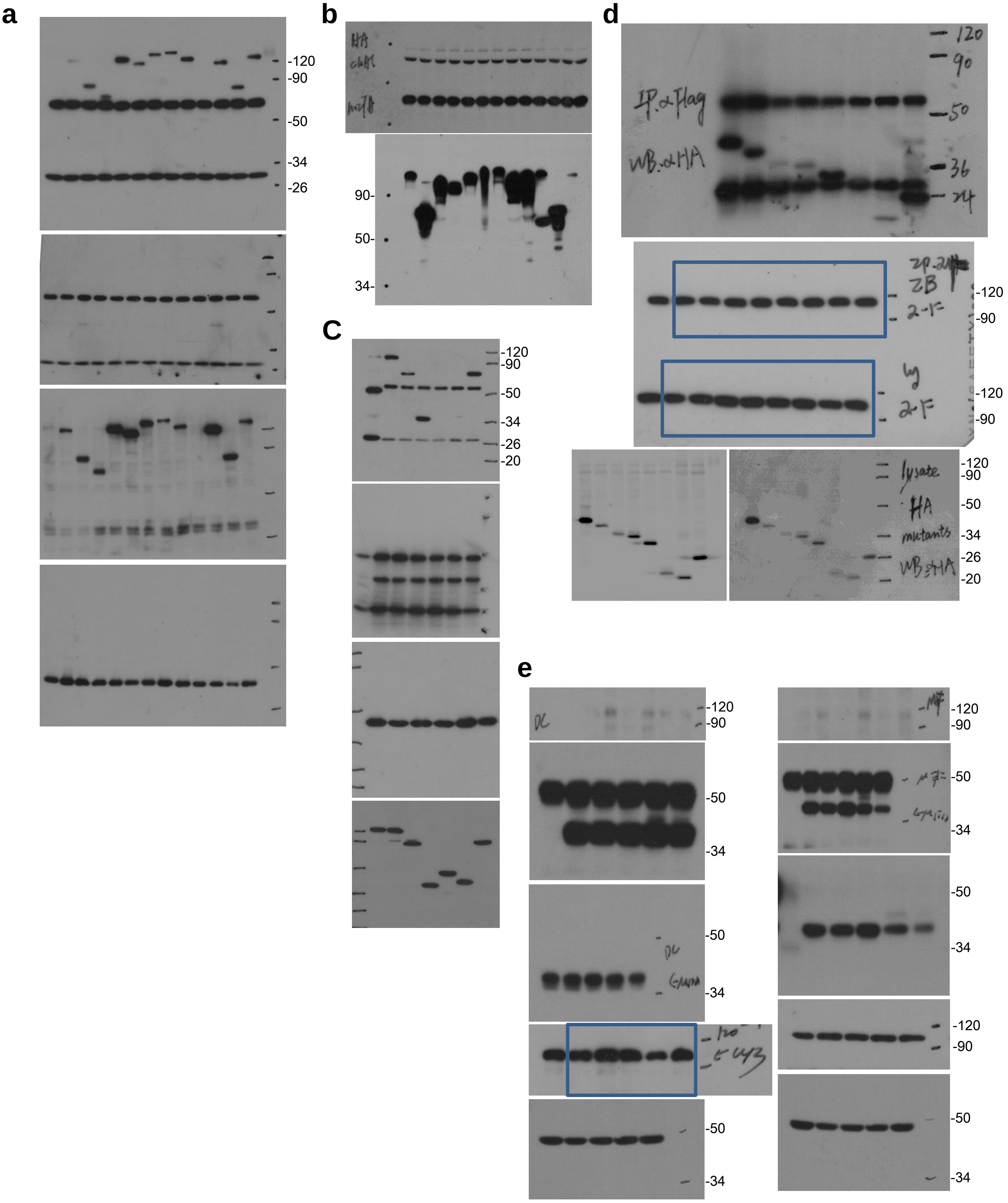

Supplementary Figure 12 Original immunoblots for Figures 2, 3, and 5

Figure 2

a

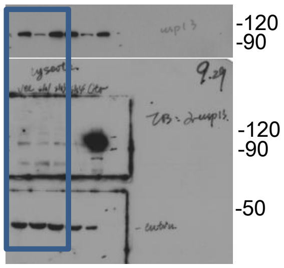

c

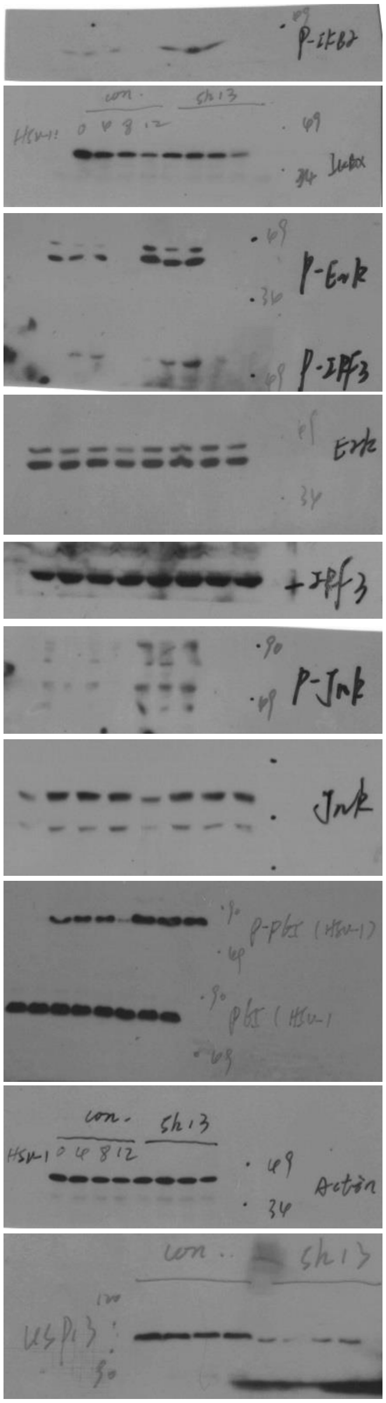

Figure 3

c

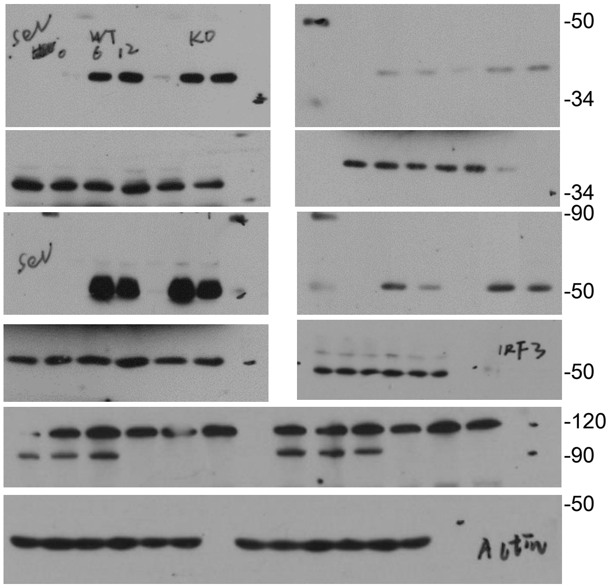

Figure 5

a

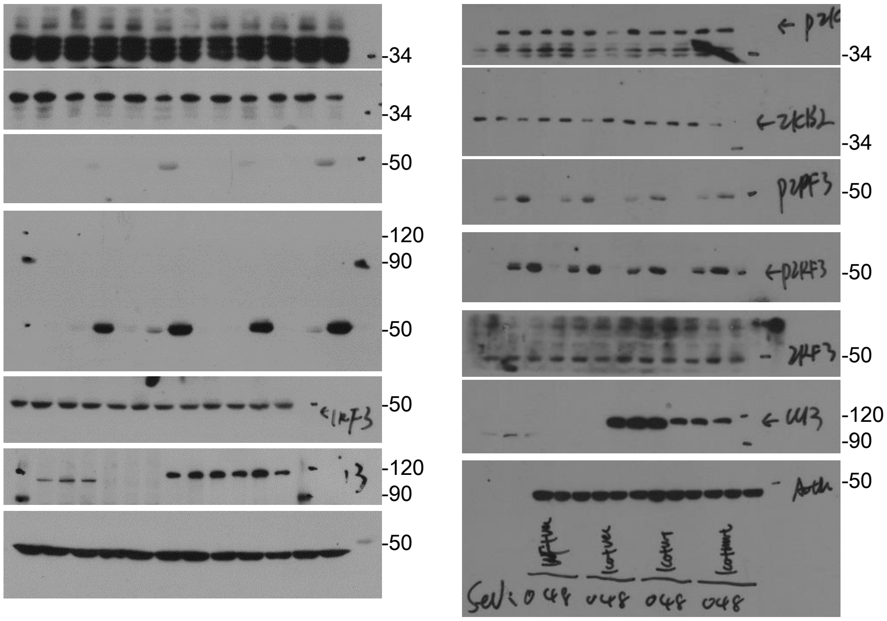

Supplementary Figure 13 Original immunoblots for Figures 6 and 7

Figure 6

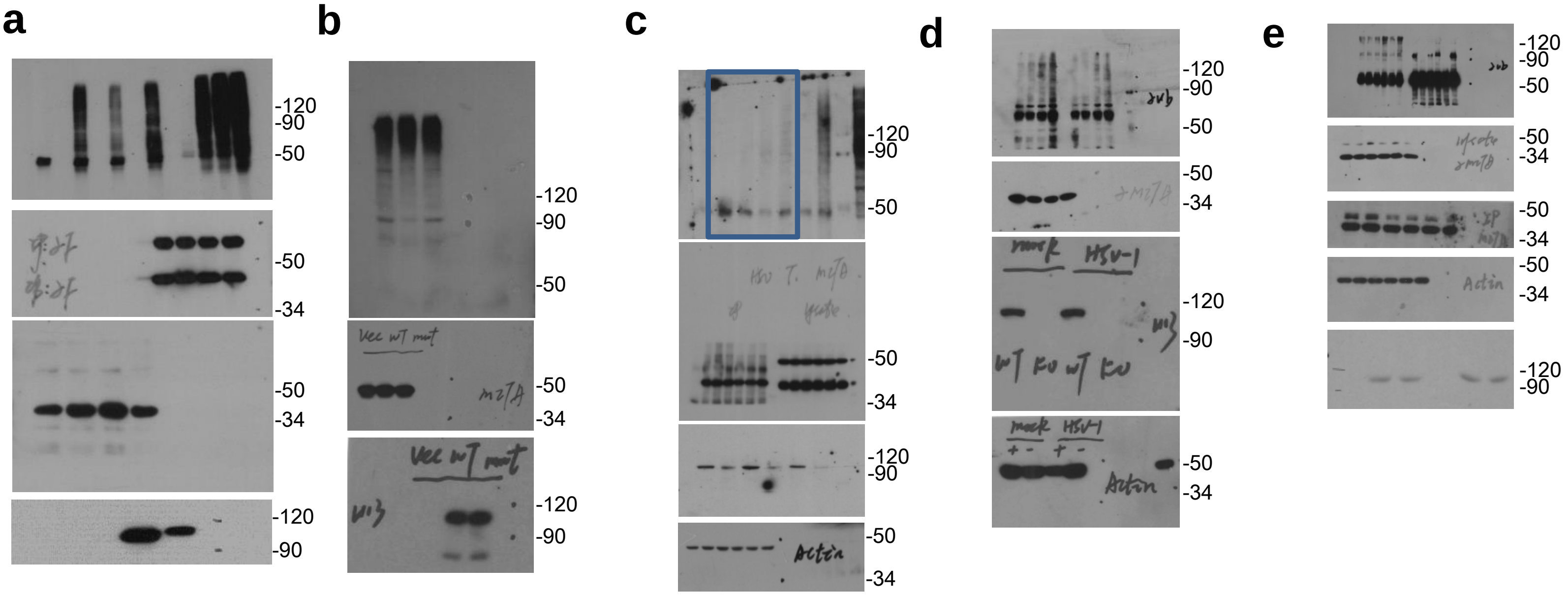

Figure 7

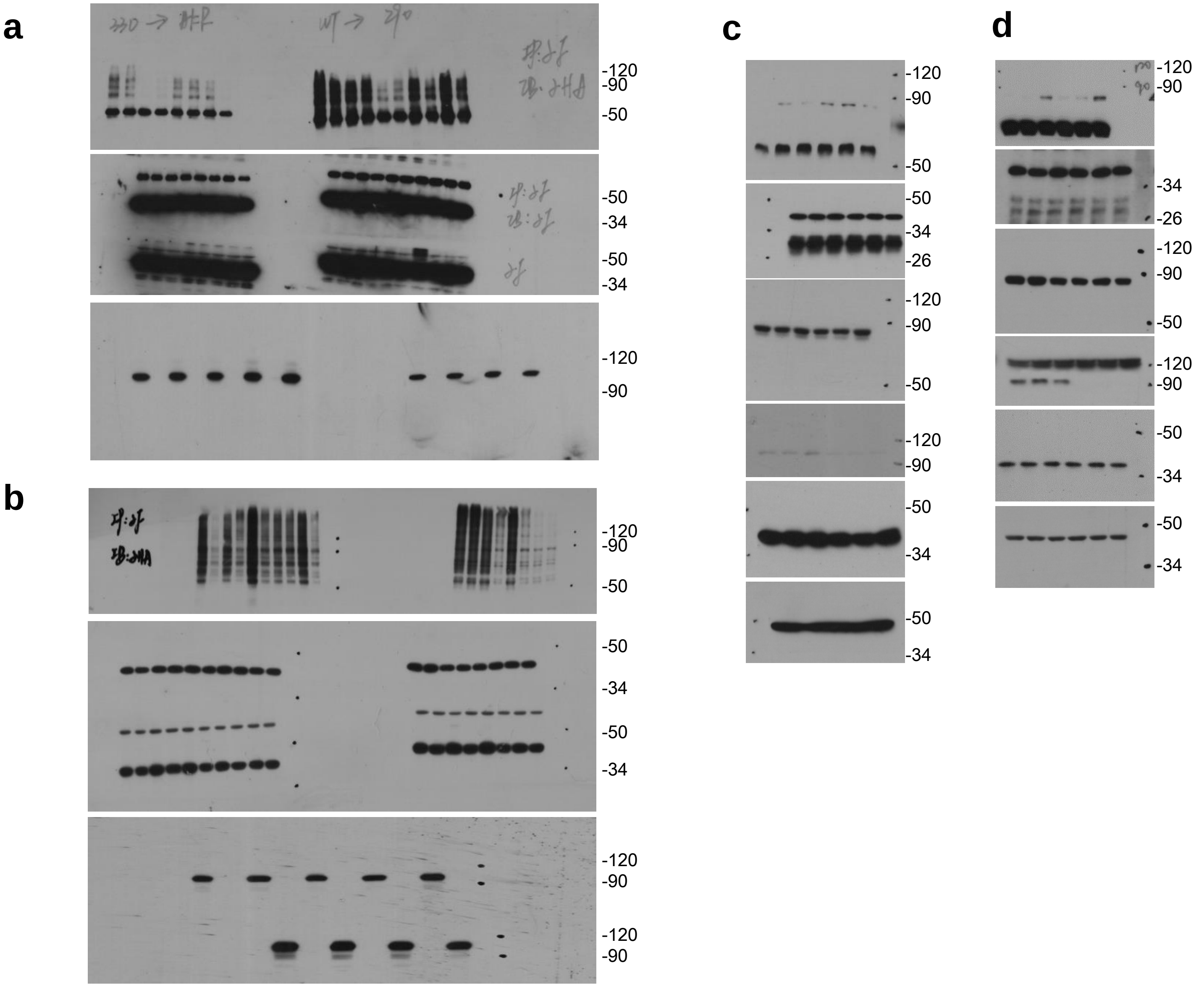

Supplement: Supplementary Information — Supplementary Figures [file ncomms15534-s1.pdf]
